# Structural Compatibility and Dynamic Behaviour of Elsholtzia Ketones in the CCT128930-Defined TRPM7 Vanilloid-like Region

**DOI:** 10.3390/ph19101530

**Published:** 2026-09-27

**Authors:** Jonas Jurevičius, Irma Martišienė, Mantė Almanaitytė, Inga Andriulė, Regina Mačianskienė

**Affiliations:** Laboratory of Membrane Biophysics, Institute of Cardiology, Lithuanian University of Health Sciences, Sukilėlių Ave. 15, LT50162 Kaunas, Lithuania; jonas.jureviciusjj@gmail.com (J.J.); irma.martisiene@lsmu.lt (I.M.); mante.almanaityte@lsmu.lt (M.A.); inga.andriule@lsmu.lt (I.A.)

**Keywords:** TRPM7, *Elsholtzia ciliata*, Elsholtzia ketone, dehydroelsholtzia ketone, molecular docking, vanilloid-like region, CCT128930, molecular dynamics

## Abstract

**Background/Objectives**: Transient receptor potential melastatin 7 (TRPM7) is a multifunctional ion channel-kinase containing a structurally resolved vanilloid-like (VL) regulatory region. Elsholtzia ketone (EK) and dehydroelsholtzia ketone (DEK), major ketones of *Elsholtzia ciliata* essential oil, are associated with cardiovascular effects, but their molecular target spectrum remains incompletely defined. We investigated whether EK and DEK can access and maintain structurally plausible configurations within the CCT128930-defined TRPM7 VL region. **Methods**: The 2.45 Å cryo-EM structure of mouse TRPM7 bound to CCT128930 (PDB 8W2L; M05) was prepared in an explicit lipid membrane and subjected to restrained minimization. The workflow combined repeated M05 redocking, blind and focused docking, RICS-based within-ligand pose selection, pose clustering, property-matched decoy controls, and 100-ns explicit-membrane molecular dynamics (MD). **Results**: Minimization preserved the VL architecture and all 12 M05 reference contacts. Ten independent M05 redocking runs recovered native-like poses in every run (best direct heavy-atom RMSD, 0.567 Å), although these ranked only 8–10 by Vina energy. Blind docking independently sampled the VL region for M05, EK, and DEK. Focused docking identified recurrent EK and DEK pose families reproducing 10/12 M05 contacts (Jaccard = 0.833). Property-matched decoys reached comparable high-end contact similarity, showing that reference compatibility was not ligand-specific. During MD, M05 showed the strongest positional and contact retention, EK intermediate redistribution, and DEK greater copy-dependent heterogeneity. **Conclusions**: EK and DEK can adopt M05-compatible geometries within the TRPM7 VL region, providing experimentally testable structural hypotheses. However, docking similarity and RICS do not demonstrate affinity, selectivity, potency, or functional TRPM7 modulation.

## 1. Introduction

Transient receptor potential melastatin 7 (TRPM7) is a widely expressed member of the TRP melastatin family and is unusual among mammalian ion channels because it combines a non-selective cation channel highly permeable to divalent cations with a C-terminal serine/threonine α-kinase within a single protein [1,2,3,4]. This dual channel-kinase architecture has led to its description as a “chanzyme” and enables TRPM7 to couple ion permeation to intracellular signalling. TRPM7 contributes to Mg^2+^, Ca^2+^, and Zn^2+^ homeostasis and participates in cell proliferation, differentiation, migration, survival, cytoskeletal regulation, and multiple signalling processes [1,2,3,4,5]. TRPM7 is expressed in the cardiovascular and nervous systems, kidney, immune cells, and numerous tumour types, consistent with broad physiological and pathophysiological functions [1,2,3,4,5,6,7].

In the cardiovascular system, TRPM7 has been implicated in vascular and endothelial function, fibrosis, myocardial remodelling, cardiac conduction, and electrical activity. Native TRPM7-like current has been characterized electrophysiologically in human atrial myocytes [8], while Mg^2+^-sensitive TRPM6/7-like currents were described earlier in cardiac myocytes [9] and were subsequently shown to influence cardiac action-potential configuration [10]. At the expression level, TRPM6 and TRPM7 are co-expressed in cardiomyocytes from all four chamber walls of the human heart [11], and both proteins have been detected in normal and diseased atrial tissue [12]. Human cardiac TRPM7 current is also strongly regulated by extracellular pH and divalent-cation concentrations [13]. These observations place TRPM7 within a broader network of ion-channel mechanisms relevant to cardiac excitability and cardiovascular regulation.

Recent cryo-electron microscopy has transformed the structural pharmacology of TRPM7 by providing ligand-bound templates for direct analysis of regulatory sites [14,15]. Functional TRPM7 is a homo-tetrameric channel in which each subunit contains six transmembrane helices (S1–S6). The S5–S6 regions from the four subunits form the central ion-conduction pore, whereas the S1–S4 region lies peripherally and is structurally coupled to the pore domain through the S4–S5 linker and the cytoplasmic TRP helix. Structures with chemically distinct inhibitors revealed a membrane-embedded regulatory region formed by residues from S3, S4, the S4–S5 linker, and the TRP helix [14,15]. Because this pocket is peripheral to the conduction pathway, ligand action at this site can be allosteric rather than pore-occluding.

TRPM7 belongs to the melastatin (TRPM) subfamily of transient receptor potential channels; it is not a member of the vanilloid (TRPV) subfamily. The term “vanilloid-like” used here refers exclusively to the topological position of the regulatory pocket which corresponds to the canonical vanilloid-binding region in TRPV channels. Thus, it does not imply that TRPM7 is a TRPV channel or that its ligands are chemically vanilloid compounds.

The CCT128930-bound TRPM7 structure is especially useful as an experimental reference. In PDB 8W2L, CCT128930 (chemical-component identifier M05) occupies the VL region at the interface between the S1–S4 and pore domains, immediately above the TRP helix [15]. Principal structural contacts involve the S3 region, the C-terminal portion of S4, the S4–S5 linker and the TRP helix. CCT128930 does not act as a classical pore blocker; the ligand-bound structure is associated with stabilization of a closed, non-conducting conformation [15]. In the apo channel, an endogenous lipid acyl chain occupies the corresponding region in the apo channel and is displaced in inhibitor-bound structures [14,15], emphasizing the close relationship between this regulatory site and the membrane environment.

Growing interest in TRPM7 biology has stimulated the search for small-molecule modulators. A chemically diverse set of compounds can inhibit or modulate TRPM7, but many show incomplete selectivity, concentration-dependent effects, or activity at other ion channels [1,2,3,4,5,6,7]. The commonly used modulator 2-aminoethoxydiphenyl borate (2-APB) is neither selective for TRPM7 nor mechanistically restricted to direct occupation of a single site; intracellular acidification contributes to its inhibition of TRPM7 [16]. Waixenicin A is a natural diterpenoid with well-established magnesium-dependent functional inhibition of TRPM7 [17]. Carvacrol is also relevant because direct patch-clamp experiments identified it as an inhibitor of mammalian TRPM7 channels [18]; subsequent studies linked TRPM7 inhibition by carvacrol to protective or antitumor effects [19,20,21]; and carvacrol inhibits native TRPM7-like current in human atrial myocytes [8] and modifies cardiac electrical activity in multicellular cardiac preparations [22]. These compounds provide useful pharmacological context, but functional modulation alone does not demonstrate occupation of the CCT128930-defined VL site.

The principal compounds of interest in the present study are EK and DEK, two major ketone constituents of *Elsholtzia ciliata* essential oil (EO). Their inclusion is supported by previous experimental cardiovascular observations rather than by structural resemblance to CCT128930. In isolated rabbit heart preparations, *Elsholtzia ciliata* EO produced concentration-dependent depression of action potential upstroke, slowed conduction, and use-dependent effects consistent with preferential inhibition of cardiac Na^+^ conductance and a Class IB antiarrhythmic phenotype [23,24]. Experiments in swine demonstrated additional cardiac electrophysiological and systemic haemodynamic effects [24], and independent work showed smooth muscle relaxation [25]. Because these experiments used EO rather than purified EK or DEK, they do not establish that either individual ketone is responsible for the observed effects. They do, however, provide a biological rationale for investigating the major ketone constituents across molecular systems relevant to cardiovascular function.

Two simplified furan-containing compounds, 2-furyl methyl ketone (2-FMK, 2-acetylfuran) and 5-methylfuran-2-carbaldehyde (5-MF), were included to probe the contribution of the furan-containing scaffold. Both retain a furan ring, whereas 2-FMK also contains a ketone functionality. Their inclusion is additionally supported by recent in vivo observations of acute hypotensive effects [26]. Carvone and carvacrol provide a complementary natural compound comparison. The comparison was used to assess whether the broader EK and DEK molecular architecture contributes to the ability to reproduce the M05-defined interaction environment, rather than attributing such behaviour to a single furan or carbonyl motif. The chemical structures and the rationale-based grouping of the complete compound panel are summarized in Figure 1.

The previously observed Class IB-like electrophysiological phenotype of *Elsholtzia ciliata* EO also provides a rationale for comparison with established Class I antiarrhythmic drugs. Lidocaine and mexiletine represent Class IB Na^+^-channel blockers, quinidine a Class IA drug, and flecainide a Class IC drug [27]. These drugs are included as pharmacologically informative structural comparators, not as established TRPM7 ligands. VER155008, NS8593, and Waixenicin A provide TRPM7-related structural or functional context, whereas 2-APB is treated separately because its functional modulation does not establish experimental occupancy of the M05-defined VL region. Figure 1 places these chemically and pharmacologically distinct groups in a single framework before the structural analyses.

Molecular docking can generate plausible ligand orientations and approximate energetic rankings, but scoring functions incompletely represent receptor flexibility, solvation, entropy, and the membrane environment [28,29,30,31,32]. Consequently, the energetically top-ranked docking pose does not necessarily correspond to the experimentally observed binding geometry. Consensus and rescoring approaches have also been developed to improve pose-ranking and virtual-screening robustness [33,34,35]. Residue-level interaction fingerprints offer a complementary way to compare poses with an experimental complex [36,37,38]. The availability of the CCT128930/M05-bound TRPM7 structure makes it possible to anchor pose analysis to a real interaction environment rather than to docking energy alone.

The present study was designed as an integrated structural analysis with three related objectives. The first objective was to determine whether EK and DEK could sample the experimentally defined VL region during blind docking and form recurrent local poses compatible with the M05 contact environment. The second was to evaluate the robustness and specificity of this reference compatibility through M05 redocking, pose family recurrence, decoy controls matched by physicochemical properties, and chemically or pharmacologically motivated comparators. The third was to examine the persistence and redistribution of selected M05, EK, and DEK structural hypotheses during 100-ns molecular dynamics in an explicit membrane. This framework was used to generate experimentally testable structural hypotheses rather than to infer binding free energy, pharmacological potency, selectivity, or functional TRPM7 modulation.

## 2. Results

### 2.1. Overview of the Computational Workflow

The analysis followed a sequential validation chain anchored to the experimentally resolved M05-bound TRPM7 structure. Structural preservation and M05 redocking were evaluated first. Blind docking then tested whether the VL region could be recovered without a search centered on M05. Focused docking provided dense local sampling, and RICS was used to select one representative pose within each ligand ensemble. Pose-family recurrence and physicochemically matched decoy controls were then assessed, followed by a separate structural analysis of 2-APB and explicit-membrane molecular dynamics. Figure 2 summarizes the workflow and the specific question addressed at each stage.

### 2.2. Preservation of the Experimental M05-Defined VL Architecture During Membrane Preparation and Minimization

The experimental 8W2L structure provided a stable starting point for the computational analysis. Restrained minimization in the explicit membrane produced only minimal displacement of the resolved protein architecture. Across 3948 common Cα atoms, the global Cα RMSD between experimental and minimized structures was 0.0485 Å; backbone and heavy-atom RMSD values were 0.0628 and 0.1135 Å, respectively. The VL region was even more strongly conserved, with Cα, backbone, and heavy-atom RMSD values of 0.0351, 0.0395, and 0.0864 Å. After protein alignment, the M05 heavy-atom RMSD was 0.0918 Å, and all 12 residues in the geometric M05 contact fingerprint remained in contact. Thus, the membrane preparation and restrained relaxation preserved the experimentally resolved M05/VL structural environment at both global and local levels.

The structural reference and its location within the tetramer are shown in Figure 3. The pocket is peripheral to the central pore and spans elements of S3, S4, the S4–S5 linker, and the TRP helix. This experimentally preserved environment was used for all subsequent redocking, contact-fingerprint, and MD comparisons.

### 2.3. Repeated M05 Redocking Recovers the Experimental Geometry but Vina Energy Does Not Rank It First

Repeated M05 redocking separated two questions that are often conflated in docking validation: whether the experimental pose can be generated and whether the scoring function ranks that pose first. Native-like M05 geometries (direct heavy-atom RMSD ≤ 2.0 Å) were recovered in all 10 independent Vina runs. The best recovered pose had an RMSD of 0.567 Å and occurred in Run 8 at Vina rank 10 with a score of −8.147 kcal mol^−1^. Across the 10 runs, the best native-like solution appeared at ranks 8–10 (median 9). Thirteen of the 1000 prospectively analysed poses (1.30%) were close to the native pose.

These results show that the protocol reproducibly generated the experimental geometry, but Vina energy ranking alone would not have selected it as the top pose. The redocking experiment therefore supports the use of experimental interaction information for representative pose interpretation while cautioning against equating energetic rank with structural correctness. The structural preservation and redocking results are summarized in Figure 4.

### 2.4. Blind Docking Independently Samples the VL Region for M05, EK and DEK

Blind docking was next used to determine whether the VL region emerged without a ligand-centred search box. For M05, 23 of 399 valid poses were located within 6 Å and 96 of 399 within 10 Å of the nearest symmetry-equivalent experimental M05 centroid. The closest M05 solution showed a centroid displacement of only 0.373 Å and a direct mapped heavy-atom RMSD of 0.472 Å, independently confirming that the experimental region can be recovered from broader transmembrane searches.

EK and DEK also sampled the same region. For EK, 22 of 399 poses were within 6 Å and 63 of 399 were within 10 Å of the nearest M05 centroid. For DEK, 43 of 399 poses were within 6 Å and 82 of 399 were within 10 Å; notably, the rank-1 pose from each of the four blind-docking tiles was within 6 Å of an M05-equivalent centroid. Among VL-localized blind solutions, EK reached a best M05-contact Jaccard similarity of 0.833, and DEK also reached 0.833. These results do not establish preferential biological binding to the VL site, but they show that both ketones can reach the experimentally defined region without a focused M05-centered search constraint. A consolidated validation and sampling summary for the revised workflow is provided in Supplementary Table S3. The four blind-search regions and the spatial recovery results for M05, EK, and DEK are shown in Figure 5.

### 2.5. Focused Docking Identifies Recurrent M05-Compatible EK and DEK Pose Families

After independent VL localization, focused docking was used to characterize local pose ensembles at higher sampling density. The selected EK representative was run5_rank13, with RICS 0.816, Jaccard and Recovery values of 0.833, 10 shared M05 contacts, and no additional contacts. The selected DEK representative was run5_rank9, with RICS 0.824, Jaccard and Recovery values of 0.833, 10 shared contacts, and no additional contacts. Both compounds lacked SER921 and TYR923 from the 12-residue reference fingerprint while retaining ASP922, PHE924, ASP978, ALA981, VAL982, MET991, TRP1111, GLN1114, ARG1115 and PHE1118.

The selected EK pose had a Vina score of −5.717 kcal mol^−1^ and the DEK pose −5.878 kcal mol^−1^. These moderate values were not used to infer affinity. Instead, the principal structural observation was convergence on a common ten-residue reference contact core. PLIP analysis further showed that the selected docking poses were dominated by hydrophobic interactions. EK produced five hydrophobic contacts involving four unique residues (VAL982, GLN1114, ARG1115 and PHE1118, with two contacts to PHE1118), whereas DEK produced four hydrophobic contacts involving PHE924, VAL982 and PHE1118. No PLIP-defined hydrogen bonds were identified in these static representative poses. The corresponding three-dimensional poses and PLIP-classified interaction diagrams are shown in Figure 6.

### 2.6. RICS Selection Is Robust to Modest Energetic Reweighting, Whereas Decoys Define a Clear Specificity Limit

RICS sensitivity analysis was performed in the locked nine-ligand focused-docking dataset. With A = 0.05, all 9/9 representative poses were retained; the mean Top-10 overlap was 9.56/10, the Top-20 overlap was 20.00/20, and Spearman rank correlation was 0.9899. At the selected A = 0.10 formulation, all 9/9 representative poses were again retained, with mean Top-10 overlap 9.56/10, Top-20 overlap 19.00/20, and Spearman correlation 0.9716. A = 0.15 also retained all 9/9 representatives, whereas A = 0.20 and 0.25 retained 8/9. Thus, A = 0.10 provides a limited Vina-derived ranking contribution without changing any representative pose selected by the J-dominated interaction-pattern criterion, while preserving high agreement among upper-ranked pose sets (Supplementary Table S2).

Pose-family clustering showed that the selected EK family contained 31 of 998 focused-docking poses and was sampled in all 10 independent runs. The selected DEK family contained 21 of 997 poses and was likewise sampled in all 10 runs. Across the locked nine-ligand set, eight of nine selected families were observed in all 10 runs; Waixenicin A was observed in six. These recurrence values indicate stochastic reproducibility of local pose families under repeated docking, not equilibrium populations.

The decoy analysis provided an essential counterpoint. Across 7902 valid property-matched decoy poses, the mean Jaccard similarity to the M05 fingerprint was 0.2645 and the median 0.1875, but the upper tail extended to values comparable with the ketones: the 95th percentile was 0.750, the 99th percentile 0.8333 and the maximum 0.9167. Therefore, although EK and DEK form recurrent poses compatible with the experimental M05 interaction pattern, high reference-contact similarity is not unique to these compounds. RICS is consequently useful for selecting a representative M05-like pose within a ligand ensemble but cannot serve as a ligand-level specificity or binder/non-binder classifier. Pose-family recurrence and the decoy Jaccard distribution are summarized in Figure 7.

### 2.7. Chemically and Pharmacologically Distinct Comparators Reveal Broad Structural Permissiveness of the VL Environment

The broader comparator panel reinforced the view that the M05-defined environment can accommodate chemically diverse structures. Among TRPM7-related functional or structural comparators, the selected NS8593 pose reproduced all 12 M05 contacts without extras (RICS 0.904), VER155008 reproduced all 12 with one additional contact (RICS 0.856), and Waixenicin A reproduced all 12 with one additional contact (RICS 0.831). These results are interpreted as structural comparisons only: M05/CCT128930 is the experimentally resolved VL-site reference, whereas the functional activity of a comparator does not by itself establish the same experimental binding site. The full reference interaction compatibility summary for RICS-selected focused docking poses is provided in Supplementary Table S1.

The natural/simplified comparator series showed graded reference compatibility. Carvacrol reproduced 10/12 M05 contacts (RICS 0.770), carvone 9/12 (RICS 0.762), and the smaller 2-FMK and 5-MF analogues each reproduced 8/12 contacts (RICS 0.687 and 0.700, respectively). The simplified compounds therefore retained part of the VL contact core but lost more of the reference environment than EK and DEK. This pattern is consistent with a contribution from overall molecular dimensions, hydrophobic surfaces, and substituent arrangements rather than from the furan ring alone.

The Class I antiarrhythmic comparators also produced M05-compatible selected poses: lidocaine reproduced 12/12 contacts without extras (RICS 0.968), mexiletine 10/12 without extras (RICS 0.850), flecainide 12/12 with one additional contact (RICS 0.864), and quinidine 12/12 without extras (RICS 0.900). These findings are not evidence that TRPM7 is a pharmacological target of these drugs. Their value is comparative: compounds chosen because of the earlier Class I-like electrophysiological context can occupy overlapping structural space in the modeled VL region, illustrating the chemical permissiveness of the cavity and further underscoring why contact similarity cannot be equated with target specificity.

Supplementary Figure S1 compares these ligand groups in a common structural frame. Supplementary Figures S2–S4 provide detailed structural and PLIP analyses, while Supplementary Figure S6 summarizes the static residue contact fingerprint across selected docking poses. The latter should be distinguished from the dynamic contact occupancies obtained from molecular dynamics trajectories.

### 2.8. 2-APB Forms a Recurrent M05-Compatible Pose Family in the Focused Region but Does Not Establish VL-Site Preference

The separate AutoDock4 analysis of 2-APB generated 1000 poses from 10 runs. Across the full ensemble, mean Recovery was 0.7734 and mean Jaccard similarity 0.7006, with a maximum of 0.8333. The representative run1_model30 pose reproduced 10/12 M05 contacts (Recovery = Jaccard = 0.8333) without additional contacts. Clustering produced 11 pose families; the representative family contained 629/1000 poses (62.9%) and was present in all 10 runs. The corresponding structural analysis is shown in Supplementary Figure S5, and the across-ligands contact fingerprint heatmap is shown in Supplementary Figure S6.

Because this experiment deliberately sampled the M05-centered region, the result shows that 2-APB can adopt a reproducible M05-compatible configuration when the search is restricted to that environment. It does not show that the VL region is the preferred or functionally relevant 2-APB binding site. The AutoDock4 diagnostic energy of −6.72 kcal mol^−1^ was not compared with Vina energies, and no RICS value was assigned.

### 2.9. Explicit Membrane MD Separates Persistent M05 Behaviour from Intermediate EK and Heterogeneous DEK Dynamics

Each TRPM7 tetramer contained four symmetry-related copies of the same ligand, designated HETA, HETB, HETC, and HETD. These copies represent four ligand instances within a single tetrameric simulation and were treated as internal observations rather than independent MD replicates. The 100-ns explicit-membrane simulations provided a dynamic test of the selected structural hypotheses. Protein Cα RMSD remained compatible with a stable membrane-embedded protein framework. In the M05 system, the mean protein Cα RMSD was 3.179 Å, with a final-20-ns mean of 3.415 ± 0.122 Å. The ligand copies remained comparatively close to their initial configurations: final-20-ns heavy-atom RMSD values were 2.573 ± 0.573, 2.361 ± 0.296, 4.428 ± 0.364, and 2.027 ± 0.242 Å for HETA–HETD, respectively. Initial-site contact retention during the final 20 ns remained 63.8%, 89.6%, 71.9%, and 65.5%. M05 therefore provided the most consistently retained local reference environment among the three systems. Quantitative ligand-instance-resolved values are summarized in Supplementary Table S4.

EK displayed intermediate behaviour. Final-20-ns ligand RMSD values were 8.346 ± 1.286 Å for HETA, 3.935 ± 1.137 Å for HETB, 8.478 ± 1.560 Å for HETC, and 3.934 ± 0.735 Å for HETD. Corresponding final-20-ns initial-site contact-retention values were 34.7%, 61.7%, 23.1%, and 66.2%. Importantly, the higher RMSD copies did not simply behave as featureless dissociated ligands; residue-level analysis showed redistribution into alternative local contact environments. For example, EK HETA developed late contacts with TYR923, LEU977 and GLY994, while HETC developed strong late contacts with PHE924 and TYR923. HETD formed late contacts with TRP1111, ARG1115, ASP978 and VAL982. Thus, EK dynamics were heterogeneous but often remained associated with the broader local transmembrane interaction environment.

DEK showed the greatest heterogeneity. HETA retained a moderately displaced local configuration (final-20-ns RMSD 5.739 ± 1.128 Å; retention 55.6%), whereas HETB, HETC and HETD reached final-20-ns RMSD values of 19.281 ± 0.455, 15.344 ± 1.235 and 14.593 ± 0.425 Å, with final-20-ns initial-site contact-retention values of 0.0%, 0.1% and 0.0%, respectively. Residue-level analysis again showed structured redistribution rather than allowing a simple binary “bound/unbound” classification. DEK HETB developed late contacts with VAL974, ILE970 and LEU977; HETC contacted MET906, GLU1124, LYS1125, GLU903 and several additional residues; HETD formed strong late contacts with ILE993, LEU977, VAL974 and TYR923. The selected docking pose was therefore not dynamically retained uniformly across the four symmetry-related DEK copies.

The three systems consequently formed a qualitative structural-dynamic hierarchy: M05 showed the strongest preservation of the initial interaction environment, EK showed intermediate local redistribution with copy-dependent retention, and DEK showed the most extensive rearrangement. Because each ligand system was represented by one tetrameric simulation, HETA–HETD are internal symmetry-related observations rather than independent replicates. The trajectories therefore support comparative structural interpretation but do not provide statistical estimates of binding free energy, residence time, or equilibrium occupancy. Figure 8 summarizes protein stability, ligand-centroid displacement, and initial-site contact retention; ligand-instance heavy-atom RMSD is shown in Supplementary Figure S7, residue-level contact evolution in Supplementary Figure S8 and the quantitative ligand-instance summary in Supplementary Table S4.

## 3. Discussion

### 3.1. Principal Findings

This study combines an experimental structural reference with docking validation, blind localization, reference-guided pose analysis, negative controls, and molecular dynamics in an explicit membrane to examine EK and DEK in the TRPM7 VL region. Restrained minimization produced minimal changes in the experimental M05 pocket. Repeated M05 redocking showed that Vina reproducibly generated poses close to the experimental geometry, although these were not ranked first by energy. Blind docking showed that M05, EK, and DEK sampled the VL region without a search box centered on M05. Focused docking identified recurrent EK and DEK pose families that reproduced 10 of the 12 M05 reference contacts, while the decoy analysis showed that this degree of contact similarity was not specific to the ketones. The 100-ns MD simulations further distinguished the modeled systems, with M05 showing the greatest persistence, EK intermediate local redistribution, and DEK the greatest heterogeneity.

Taken together, these analyses are more informative than a docking score alone, but they also set clear limits on interpretation. The results support structural compatibility of EK and DEK with the VL environment defined by M05 and identify specific configurations for experimental testing. They do not show that EK or DEK binds TRPM7 with measurable affinity in cells, inhibits the channel, stabilizes the same conformational state as CCT128930, or accounts for the cardiovascular effects of *Elsholtzia ciliata* EO.

### 3.2. Biological Rationale for EK and DEK and the Significance of the Comparator Design

The rationale for studying EK and DEK comes from the pharmacology of *Elsholtzia ciliata* rather than from structural similarity to CCT128930. *Elsholtzia ciliata* EO alters cardiac excitability and conduction in isolated rabbit hearts and produces cardiovascular effects in swine [23,24]; it also relaxes smooth muscle [25]. The Class IB-like component of the cardiac phenotype prompted earlier interest in Na^+^ conductance and explains the inclusion of Class I antiarrhythmic drugs as comparators here. The essential oil studies, however, do not identify purified EK or DEK as the active compounds and do not implicate TRPM7 as the mediator. The present work therefore extends the earlier pharmacological observations by evaluating whether the major ketones can adopt plausible structural configurations in another ion channel system relevant to cardiovascular function.

The simpler furan analogues address a different question. EK and DEK contain a furan-based architecture, but our docking results indicate that the furan motif alone is not enough to reproduce the complete M05 reference interaction pattern. The selected EK and DEK poses retained 10 of 12 reference contacts, whereas 2-FMK and 5-MF retained 8 of 12. This difference is consistent with a contribution from the complete molecular scaffold, including molecular dimensions, hydrophobic surface, carbonyl placement, and substituent geometry. Carvone and carvacrol provide an additional comparison, showing that a pattern with 9 or 10 M05 contacts is not unique to furan ketones. Carvacrol is particularly informative because it has experimentally demonstrated TRPM7 inhibitory activity [18]. Even so, its structural similarity in the present analysis remains a comparison and does not show that all ligands compatible with M05 share the same mechanism.

### 3.3. What M05 Redocking Reveals About Docking Validation

The expanded M05 redocking experiment illustrates an important point about docking validation. A docking protocol may be able to generate an experimentally correct pose even when the scoring function does not place that pose first. In this study, poses close to the native M05 geometry were recovered in every independent run, but the best such pose appeared only at ranks 8–10. This agrees with broader docking benchmarks showing that the highest energetic rank does not necessarily correspond to the experimentally observed geometry [28,29,30,31,32]. In a system with experimental interaction data, selecting a representative pose solely because it has the lowest Vina energy would therefore be difficult to justify.

RICS was developed for a narrower task than a general docking score. Within each ligand ensemble, it selects poses that most closely reproduce the experimental M05 contact environment while giving only limited weight to Vina energy. The sensitivity analysis showed that the selected representatives remained unchanged when the energetic contribution was modest. This does not make RICS a predictor of pharmacology. It simply shows that pose selection was not dependent on one exact 0.90/0.10 weighting choice.

### 3.4. Blind Docking and Focused Docking Answer Different Structural Questions

Blind and focused docking address different questions. Blind docking asks whether the VL region can be found during a broader transmembrane search without placing the ligand directly in the experimental M05 pocket. Recovery of the region by M05 serves as a positive structural control, while localization of EK and DEK shows that the ketones can reach the same general environment without a search box centered on M05. Focused docking then asks what local pose families and contact patterns are sampled repeatedly when the search is concentrated on that region.

This distinction is especially important for 2-APB. Its focused AutoDock4 ensemble contains a large recurrent family compatible with M05. Because the search was deliberately centered on the VL region, however, this result cannot show that the VL region is the preferred 2-APB site in TRPM7. More generally, recurrence within a focused search supports geometric feasibility and reproducible sampling, but it is not independent evidence that the same site would be selected across the whole protein.

### 3.5. Reference-Contact Similarity Is Informative for Pose Interpretation but Not for Ligand Specificity

The decoy experiment is an important control because it shows what the contact-based analysis cannot establish. EK and DEK reached a Jaccard value of 0.833 against the experimental M05 fingerprint, but the 99th percentile of the decoy distribution was also 0.8333, and the maximum was 0.9167. High similarity to the M05 contact pattern can therefore occur for chemically unrelated molecules sampled in the same local cavity. For this reason, neither RICS nor Jaccard should be interpreted as evidence that a compound is a specific TRPM7 binder.

This limitation does not diminish the descriptive value of the contact analysis. Within one ligand ensemble, the experimental M05 fingerprint provides a transparent way to select and describe a pose that is compatible with the reference structure. It also allows residue-level comparison of chemically different ligands in the same structural frame. RICS is therefore best used as a descriptive tool for pose selection: it asks which sampled pose most closely resembles the experimental interaction environment, not which ligand binds best.

### 3.6. Structural Interpretation of EK and DEK Within the VL Environment

EK and DEK are closely related chemically, yet they behave differently in the TRPM7 VL environment. Their main structural difference is the additional unsaturation in DEK. Although this is a small chemical change, a double bond restricts rotation and can reduce the conformational space available to a ligand. Conformational restriction and preorganization are well-established concepts in medicinal chemistry, and relatively small structural changes can substantially alter the geometries sampled during molecular recognition [39].

The selected EK and DEK poses from focused docking were very similar in their compatibility with the reference: both reproduced 10 of the 12 M05 contacts (Jaccard = 0.833) and introduced no additional contacts. The difference became apparent when the broader pose ensembles were considered. In blind docking, 22 of 399 EK poses and 43 of 399 DEK poses were within 6 Å of the nearest symmetry-equivalent M05 centroid. This almost twofold difference indicates different spatial sampling of the VL region; it should not be interpreted as stronger DEK binding. Studies of closely related ligand series likewise show that small chemical changes can alter binding geometry, dynamics, and thermodynamic behaviour even when the change in overall affinity is modest [40].

The MD simulations provide a complementary view. M05 remained relatively constrained, EK showed intermediate local redistribution, and DEK showed greater variation among ligand copies, with substantial positional redistribution in several of them. Thus, the more frequent sampling of the VL region by DEK during blind docking did not lead to uniformly better retention of the initial interaction geometry. One possible explanation is that the greater conformational freedom of EK allows local adjustment, whereas the more constrained geometry of DEK may favour access to some VL-compatible arrangements but allow less internal adaptation as the protein and membrane fluctuate. Studies of related ligands have shown that even homologous compounds can be accommodated by different protein conformations, illustrating the close connection between ligand flexibility and binding site variability [41].

These results suggest that three features should be considered separately: access to the VL region, compatibility with the M05 reference contact environment, and dynamic retention of the resulting geometry. The calculations do not show that either ketone has higher affinity or stronger functional effects on TRPM7. Instead, they suggest a structure and dynamics hypothesis that can be tested experimentally. The different behaviour of two closely related ketones that occur together in *Elsholtzia ciliata* also raises a broader possibility: modest structural diversity in a natural mixture may allow its components to accommodate a wider range of protein microenvironments or conformational states. This remains a hypothesis. It could be tested by comparing purified EK and DEK, defined mixtures of EK and DEK, and *Elsholtzia ciliata* preparations under the same functional conditions.

### 3.7. Molecular Dynamics Reveals Different Degrees of Persistence and Local Redistribution

The MD simulations add information that cannot be obtained from static docking. M05, the experimentally resolved reference ligand, showed the greatest overall preservation of its initial local environment. Its RMSD remained comparatively low, and substantial contact retention was seen in all four symmetry-related copies during the final 20 ns. This behaviour is consistent with the experimental structural role of M05 and supports the physical plausibility of the prepared membrane system.

EK behaved differently. Two ligand copies retained relatively modest final RMSD values, whereas two underwent larger rearrangements. Contact analysis showed that these changes often involved redistribution among nearby residues rather than simple movement into bulk solvent. This distinction matters because ligand RMSD alone can overstate structural loss when a small molecule rotates or translates within an extended local cavity. Considering RMSD, centroid displacement, and contacts together, EK showed intermediate local persistence with substantial flexibility of its pose.

DEK was more heterogeneous. Three ligand copies developed large RMSD values late in the trajectory and lost almost all contacts present at frame 0, while forming new contacts in neighbouring transmembrane regions. Under the present simulation conditions, the selected DEK docking geometry was therefore less persistent than the M05 reference and less consistently retained than the EK geometry. This should not be interpreted directly as weaker binding. One 100-ns trajectory per ligand system is not sufficient to determine converged binding thermodynamics, and the four ligand copies are internal symmetry-related observations rather than independent replicates.

The MD results refine the docking interpretation by showing different degrees of positional and contact persistence among the three modelled systems. M05 was the most persistent reference, EK showed intermediate local redistribution, and DEK showed greater heterogeneity. Whether these differences relate to channel state dependence, residence behaviour, or functional coupling cannot be determined from the present simulations and requires direct electrophysiological, binding, or free energy studies.

### 3.8. Pharmacological Implications

TRPM7 is relevant to cardiovascular physiology, and EK/DEK originate from a preparation with documented cardiac and vascular actions. It is therefore reasonable to consider TRPM7 as one candidate molecular system in a broader target spectrum. The present structural results make that hypothesis more concrete by identifying an experimentally defined regulatory region that can accommodate the ketones and by showing how their local dynamics differ from the experimental M05 reference. Nevertheless, the study does not establish TRPM7 as the molecular basis of the previously observed *Elsholtzia ciliata* effects.

Direct functional experiments would be required to move from structural compatibility to pharmacology. Recombinant or native TRPM7 electrophysiology could establish whether purified EK or DEK alters current; define concentration–response relationships, reversibility, voltage dependence and Mg^2+^ dependence; and determine whether the compounds behave as inhibitors, activators or state-dependent modulators. Mutagenesis of residues highlighted by the experimental M05 structure and the present contact analyses could then test whether any functional effect depends on the VL region. Such experiments would also allow comparison with carvacrol, Waixenicin A and CCT128930 under a common functional framework.

### 3.9. Limitations

Several limitations define the scope of the conclusions. First, the experimental structural reference is mouse TRPM7; species-specific differences must be considered before extrapolating directly to human pharmacology. Second, docking uses simplified energetic models and a largely rigid receptor representation. Blind docking reduces dependence on a predefined local box but does not reproduce the full conformational landscape of the channel. Third, the 4.0 Å contact criterion is geometric and does not assign equal energetic significance to hydrophobic, polar, aromatic, or electrostatic interactions.

Fourth, RICS is deliberately reference-based. It preferentially selects M05-like poses and may undervalue a biologically relevant pose that occupies the same cavity through a different interaction network. The decoy results further demonstrate that high RICS/Jaccard similarity is not specific to true TRPM7 ligands. Fifth, 2-APB required a separate AutoDock4 workflow, so its energy cannot be compared quantitatively with Vina energies, and its focused-docking result does not establish independent site preference.

Finally, the MD analysis comprises one 100-ns trajectory per ligand system. The four symmetry-related ligand copies increase the range of local behaviours observed within each tetramer but are not independent replicates. The simulations therefore describe structural persistence and redistribution under the modelled conditions; they do not provide converged equilibrium occupancies, residence times or binding free energies. No direct TRPM7 electrophysiology, ligand-binding assay, or VL-site mutagenesis of purified EK or DEK is included in the present study. These limitations are important because the study defines structural hypotheses, not confirmed pharmacological mechanisms.

## 4. Materials and Methods

### 4.1. Structural Reference and Ligand Panel

The 2.45 Å cryo-EM structure of mouse TRPM7 bound to CCT128930 (PDB 8W2L; ligand component M05) was used as the experimental structural reference [15]. Unresolved protein regions were not modelled. The resolved protein was maintained as separate structural segments across unresolved gaps, with terminal capping during membrane-system preparation. The biological VL region was distinguished from the computational M05 reference fingerprint: “VL region” denotes the experimentally characterized membrane-embedded regulatory pocket, whereas the M05 fingerprint denotes the set of residues satisfying the uniform geometric contact definition in the experimental complex.

Using a minimum ligand–protein heavy-atom distance of 4.0 Å, the M05 reference fingerprint comprised SER921, ASP922, TYR923, PHE924, ASP978, ALA981, VAL982, MET991, TRP1111, GLN1114, ARG1115 and PHE1118. The ligand panel included EK and DEK; 2-FMK and 5-MF; carvone and carvacrol; VER155008, NS8593 and Waixenicin A; the Class I antiarrhythmics lidocaine, mexiletine, flecainide and quinidine; and 2-APB in a separate AutoDock4 workflow. M05 was used as the experimental reference and validation ligand rather than as an ordinary screening compound.

### 4.2. Receptor and Ligand Preparation

The minimized receptor and the docking ligands were prepared for AutoDock-family calculations using Meeko 0.7.1. AutoDock-compatible PDBQT files were generated from chemically defined input structures, and the prepared structures were checked before docking. Meeko provides the molecular parametrization and interoperability layer used to encode the chemical representation required for AutoDock-compatible docking workflows [42]. The final docking receptor was the minimized TRPM7 model without the bound M05 ligand; the experimental M05 coordinates were retained separately for redocking validation and reference contact analysis.

### 4.3. Explicit-Membrane Preparation and Restrained Minimization

The 8W2L complex was prepared in an explicit membrane environment using the retained CHARMM-GUI system. The membrane contained cholesterol, POPC, POPE, and POPS, with 0.15 M KCl and 105,551 water molecules. The M05 system contained 463,971 atoms. Protein and membrane components were described with CHARMM-compatible force-field parameters; M05 used ligand-specific topology and parameters, whereas EK and DEK used CGenFF-derived ligand parameters. Restrained minimization was performed in OpenMM 8.6 using L-BFGS (mini_Tol = 1.0). A post-minimization structure was saved before velocity generation or equilibration. Preservation of the experimental model was quantified by global Cα, backbone, and heavy-atom RMSD; VL-region RMSD; M05 heavy-atom RMSD after protein alignment; and retention of the 12-residue M05 contact fingerprint.

### 4.4. M05 Redocking Validation

M05 redocking was performed with AutoDock Vina 1.2.7 [28,43]. Ten independent runs used seeds 1001–1010. Although 120 modes were generated per run, the first 100 poses from each run were prospectively retained for analysis, giving 1000 poses. Docked M05 was compared with the experimental ligand by symmetry-aware chemical-graph matching and direct heavy-atom RMSD in the receptor coordinate frame without pose superposition. A native-like pose was defined as RMSD ≤ 2.0 Å. The analysis recorded native-like recovery by run, best RMSD, the Vina rank of the best native-like pose in each run, and the total fraction of native-like poses.

### 4.5. Blind Docking of M05, EK and DEK

Blind docking was used to test whether the VL region could be sampled without defining a ligand-centred M05 box. Because a single box spanning the entire membrane tetramer would create an impractically large Vina search space, four partially overlapping geometry-derived transmembrane search regions were used. These regions were defined from protein/membrane geometry rather than from EK or DEK docking results. For each ligand, valid poses from the four searches were pooled. Localization was quantified as the distance of each pose centroid to the nearest symmetry-equivalent experimental M05 centroid. For M05, direct heavy-atom RMSD was additionally calculated for the closest recovered solution. EK and DEK blind-docking poses were also compared with the 12-residue M05 fingerprint by Jaccard similarity.

### 4.6. Focused Docking and RICS Representative-Pose Selection

After the independent localization analysis, focused docking was used for dense sampling of the M05-defined region. Protein–ligand contacts were detected with the same 4.0 Å minimum heavy-atom distance criterion. For every pose, the full contact set was compared with the 12-residue M05 reference. Recovery was defined as Nshared/Nreference, while the Jaccard coefficient was Nshared/(Nreference + Npose − Nshared). Recovery therefore measures retention of the reference set, whereas Jaccard additionally penalizes contacts outside that set.

Representative poses were selected within each ligand ensemble using the Reference Interaction Compatibility Score (RICS): RICS = 0.90J + 0.10A, where J is full-contact Jaccard similarity to the experimental M05 fingerprint, and A is a limited within-ligand Vina support term normalized over a fixed 2.0 kcal mol^−1^ window relative to the best energetic pose. RICS is a reference-based pose-compatibility metric, not a classical scoring function. It was used only to select a representative pose within a ligand ensemble and was not interpreted as binding free energy, affinity, IC_50_, potency, selectivity, or biological activity.

### 4.7. RICS Sensitivity Analysis and Pose-Family Recurrence

The Vina-support contribution to RICS was varied from A = 0.05 to 0.25, with the complementary Jaccard weight adjusted accordingly. Sensitivity was assessed in the locked nine-ligand dataset comprising EK, DEK, NS8593, VER155008, Waixenicin A, lidocaine, mexiletine, flecainide and quinidine (8934 poses). Representative-pose retention, Top-10 and Top-20 overlap, and Spearman rank correlation were calculated across the tested weighting schemes. Focused-docking poses were also clustered by 2.0 Å heavy-atom RMSD in the receptor frame. The number of independent docking runs contributing to the selected pose family was used as a stochastic recurrence measure. Cluster population was not interpreted as equilibrium occupancy or binding probability. The weighting sensitivity results are reported in Supplementary Table S2.

### 4.8. Decoy Analysis with Compounds Matched by Physicochemical Properties

Eight property-matched decoy compounds were processed through the same focused contact-comparison framework to determine whether high M05-reference similarity was specific to the investigated ligand panel. In total, 7902 valid poses were obtained from 8000 attempted decoy poses. The distribution of full-contact Jaccard similarity was summarized by its mean, median, 95th percentile, 99th percentile, and maximum. This analysis was designed as a negative control for the specificity of reference-contact similarity, not as a pharmacological binder/non-binder benchmark.

### 4.9. Separate Structural Analysis of 2-APB

Because 2-APB contains boron, it was analysed in a separate AutoDock4 workflow [44] using the boron-compatible parameterization employed in the project. The focused search was centred at the M05 reference centroid (108.155, 73.872, 78.143 Å) with a 58 × 68 × 58 grid and 0.375 Å spacing. Ten runs with 100 genetic algorithm runs each generated 1000 poses. Poses were evaluated by M05-contact Recovery and Jaccard similarity and clustered at 2.0 Å. The representative structural solution was run1_model30. AutoDock4 energy was retained only as an internal diagnostic and was not compared quantitatively with Vina energies; RICS was not assigned to 2-APB.

### 4.10. PLIP Interaction Profiling and Structural Visualization

Selected poses were visualized in PyMOL 3.1.0 using a common receptor frame and publication orientation. Three-dimensional views were used to show ligand position within the VL region, while two-dimensional interaction diagrams were generated with PLIP [37] to annotate non-covalent contacts independently of RICS. Static geometric proximity in a docking pose was not treated as evidence of a dynamically persistent hydrogen bond. Comparative overlays used the same protein reference and camera orientation to facilitate direct spatial comparison across ligand classes.

### 4.11. Molecular Dynamics in an Explicit Membrane

M05-, EK- and DEK-containing TRPM7 systems were simulated for 100 ns in explicit membrane, water and ions. Production trajectories comprised 100 sequential DCD segments and 1000 analysed frames, corresponding to 0.1 ns per analysed frame and 100 ns total sampling. Each tetrameric system contained four symmetry-related ligand copies, designated HETA-HETD. These four copies were treated as internal observations within one simulation system and not as independent simulation replicates.

Trajectory analysis included protein Cα RMSD, ligand heavy-atom RMSD after global protein Cα alignment, ligand-centroid displacement, initial-site contact retention and residue-level contact occupancy. Initial-site residues were determined independently for each ligand copy from frame 0 using a minimum ligand–protein heavy-atom distance of ≤4.0 Å and were then tracked throughout the trajectory. Full trajectory values were complemented by analysis of the final 20 ns (80–100 ns). New late contacts were identified when residues absent from a copy-specific frame-0 initial site reached ≥20% occupancy during the final 20 ns. Contact occupancy and retention were interpreted as geometric measures of local structural persistence rather than binding affinity, residence time or equilibrium site occupancy.

## 5. Conclusions

Blind docking showed that EK and DEK can access the experimentally characterized TRPM7 VL region defined by CCT128930/M05. Focused docking identified recurrent pose families for both ketones, each reproducing 10 of the 12 M05 reference contacts. Repeated M05 redocking confirmed that the protocol can generate the experimental geometry, while also showing that Vina energy alone does not reliably select it. RICS provides a transparent way to choose a representative pose that resembles the M05 contact environment within each ligand ensemble. The decoy analysis, however, shows that this similarity is not specific to EK or DEK and cannot be used as evidence of binding affinity or selectivity. The MD simulations further distinguished the three ligands: M05 was the most persistent, EK showed intermediate local redistribution, and DEK was the most heterogeneous. These results provide an experimentally anchored structural basis for testing EK and DEK at the TRPM7 VL region, while keeping computational compatibility clearly separate from demonstrated pharmacological action.

## Figures and Tables

**Figure 1:**
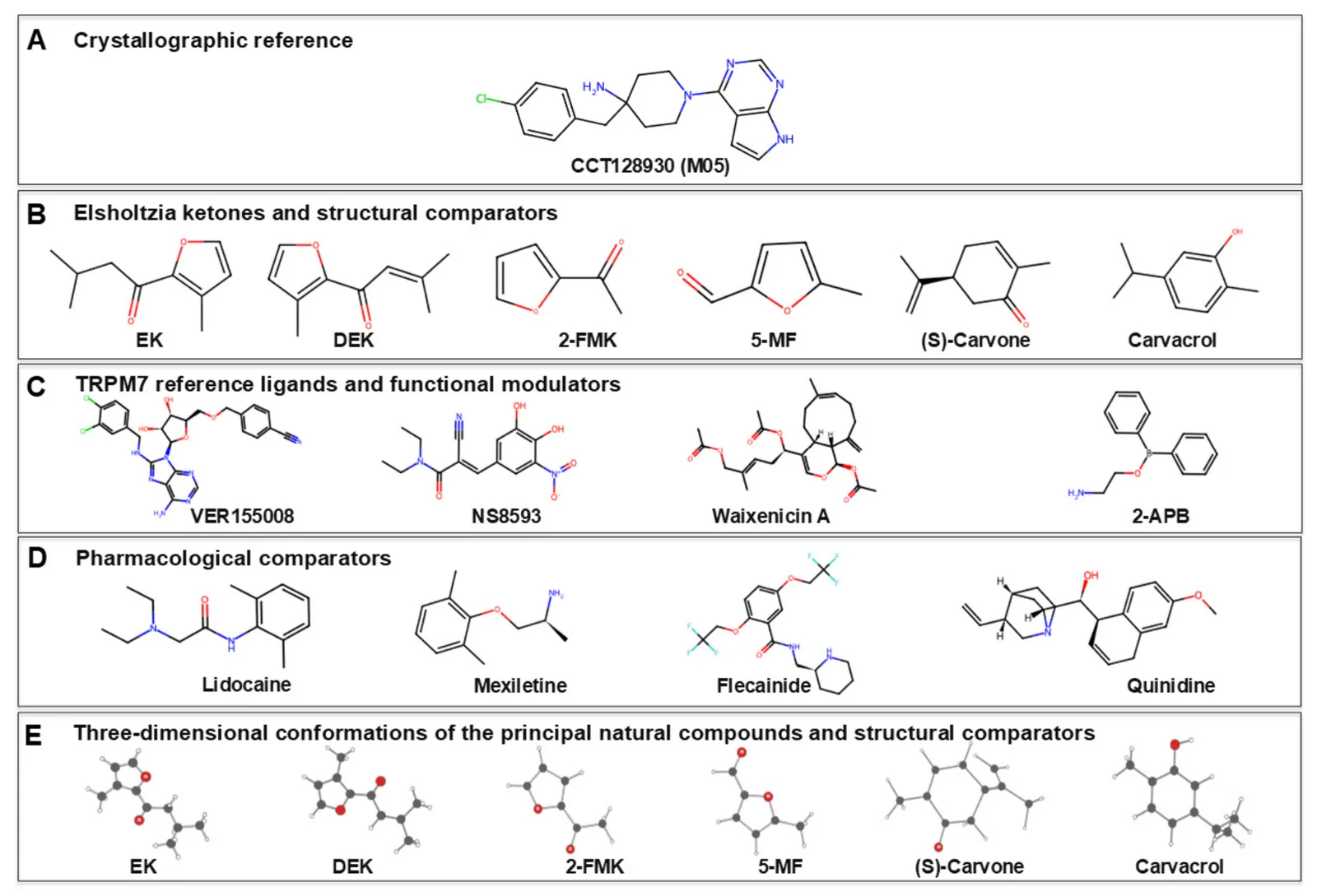
Chemical structures and compound classification. (**A**) CCT128930 (M05), the experimentally resolved ligand in TRPM7 structure 8W2L and the structural reference for the M05-defined vanilloid-like (VL) region. (**B**) EK, DEK, 2-FMK (2-acetylfuran), 5-MF, (S)-carvone and carvacrol. (**C**) TRPM7-related structural and/or functional comparators VER155008, NS8593, Waixenicin A and 2-APB; inclusion does not imply experimentally established occupancy of the M05-defined VL region. (**D**) Class I antiarrhythmics lidocaine, mexiletine, flecainide and quinidine, included as pharmacologically motivated comparators. (**E**) Three-dimensional conformations of the principal natural compounds and structural comparators.

**Figure 2:**
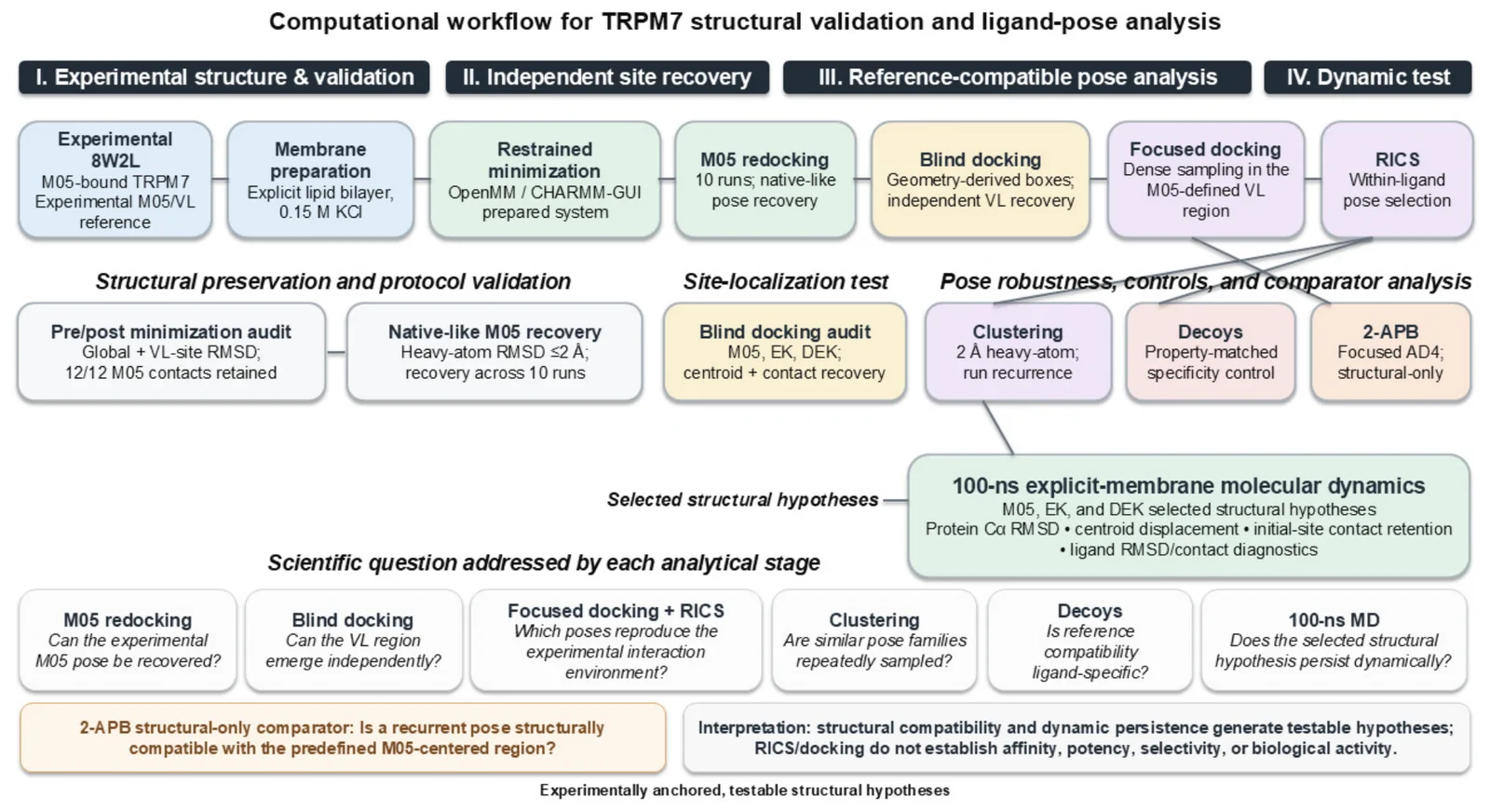
Computational workflow for TRPM7 structural validation and ligand pose analysis. The workflow progresses from the experimentally determined M05-bound TRPM7 structure (PDB 8W2L) through explicit membrane preparation and restrained minimization, repeated M05 redocking, geometry-derived blind docking, focused docking with RICS-based within-ligand pose selection, pose family recurrence and property-matched decoy controls, a separate structural 2-APB analysis, and 100-ns explicit membrane molecular dynamics. Each stage addresses a distinct question: structural preservation, experimental pose recovery, independent site localization, reference compatibility, recurrence, specificity, or dynamic persistence. These computational descriptors are not interpreted as direct measures of binding affinity, potency, selectivity, or biological activity.

**Figure 3:**
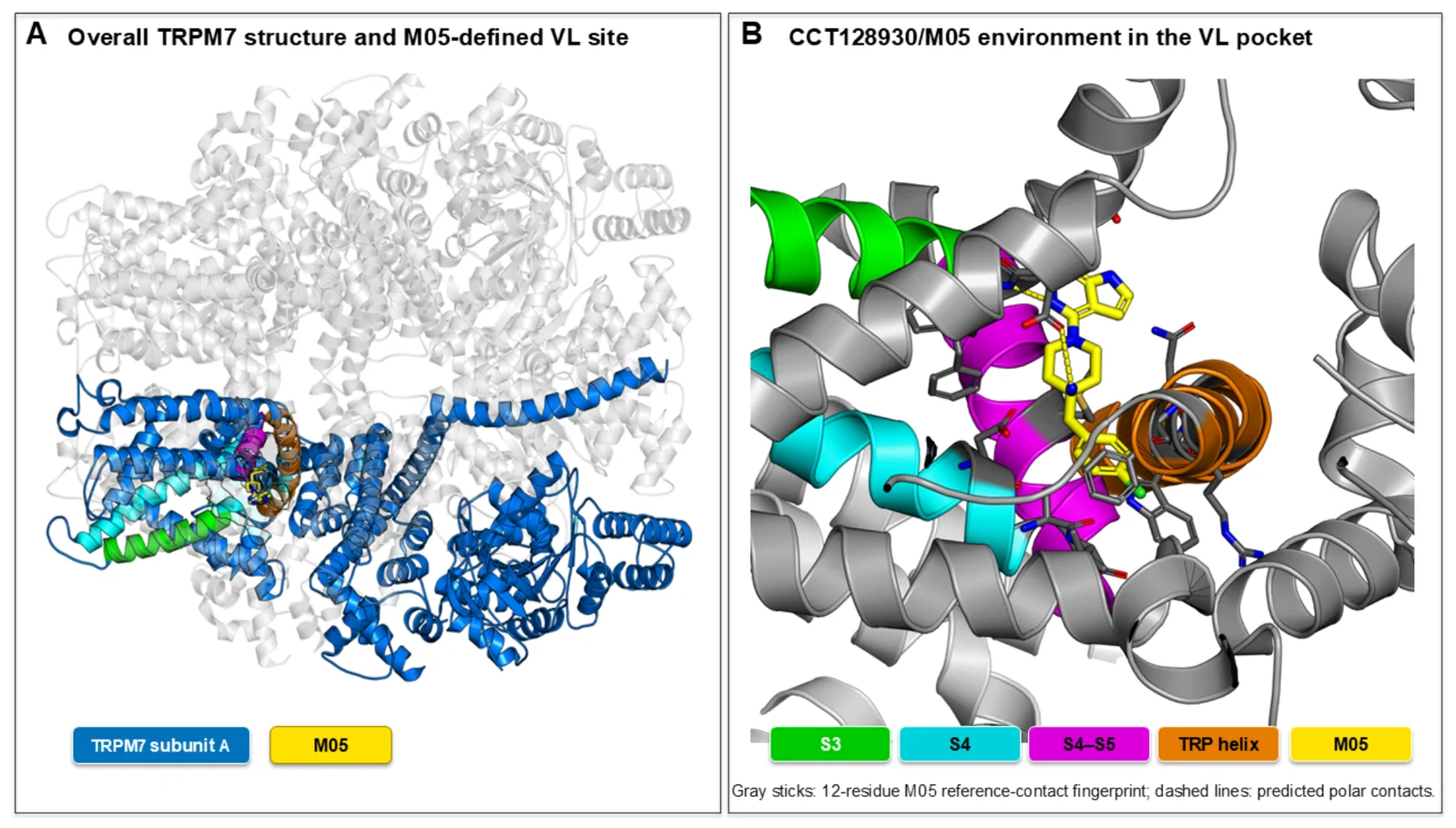
Experimental structural reference and the CCT128930/M05-defined vanilloid-like region of TRPM7. (**A**) Overall TRPM7 tetramer showing M05 within the membrane-embedded VL region; one subunit is highlighted for orientation. (**B**) Enlarged M05 environment formed by S3, S4, the S4–S5 linker, and the TRP helix. Gray sticks denote the 12-residue M05 reference-contact fingerprint (SER921, ASP922, TYR923, PHE924, ASP978, ALA981, VAL982, MET991, TRP1111, GLN1114, ARG1115 and PHE1118), defined by a minimum ligand–protein heavy-atom distance ≤4.0 Å. Dashed lines denote predicted polar contacts for geometric illustration and not dynamically persistent hydrogen bonds.

**Figure 4:**
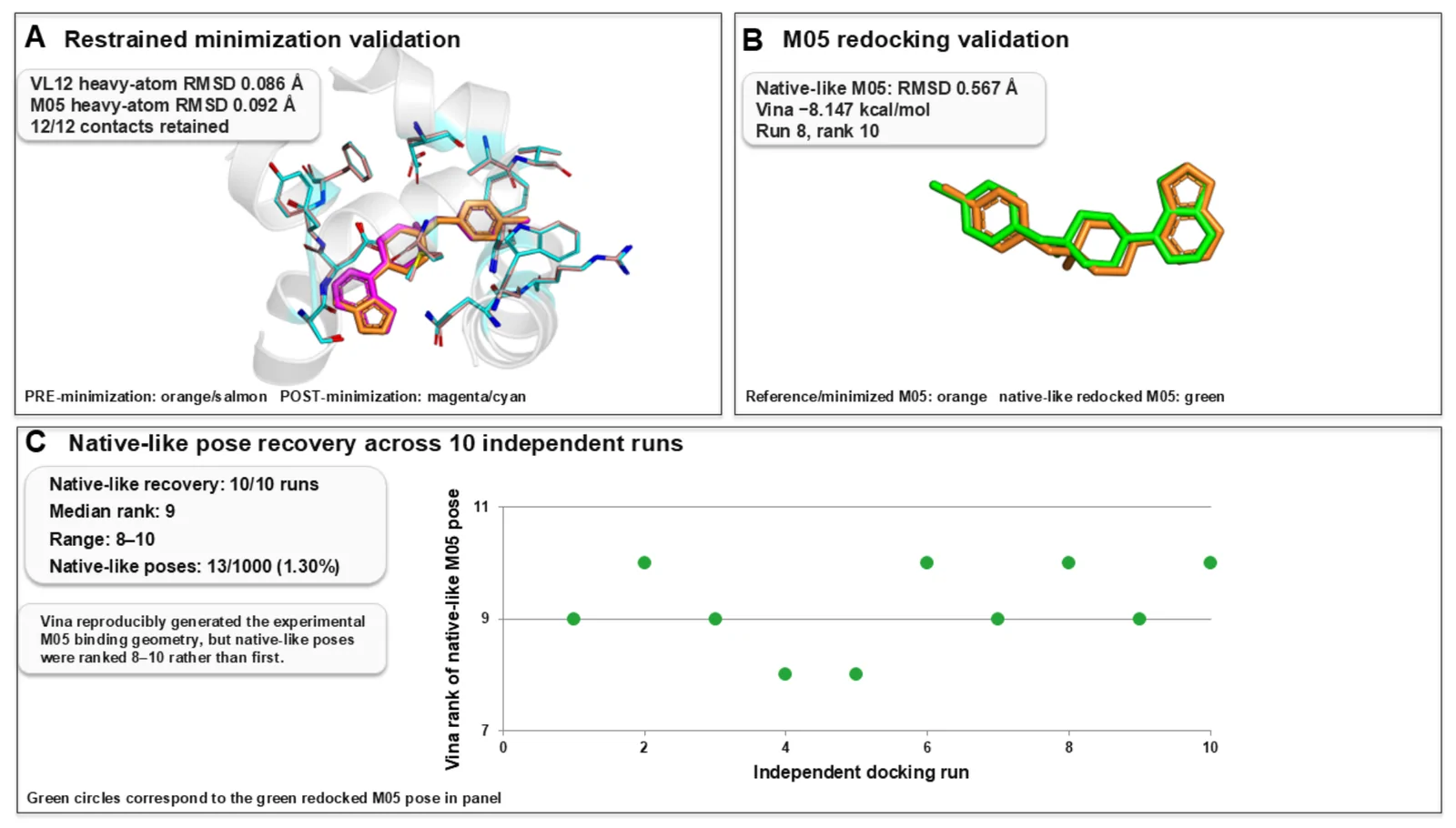
Validation of structural preservation and experimental-pose recovery in the TRPM7 computational model. (**A**) Experimental 8W2L/M05 structure before and after restrained minimization: VL12 heavy-atom RMSD 0.086 Å, M05 heavy-atom RMSD 0.092 Å, with all 12 reference contacts retained. (**B**) Experimental/minimized M05 compared with the best native-like redocked pose (direct receptor-frame heavy-atom RMSD 0.567 Å; Vina −8.147 kcal mol^−1^; Run 8, rank 10). (**C**) Best native-like rank across 10 independent Vina runs. Native-like recovery (RMSD ≤ 2.0 Å) occurred in 10/10 runs at ranks 8–10 (median 9); 13/1000 analysed poses (1.30%) were close to the native pose.

**Figure 5:**
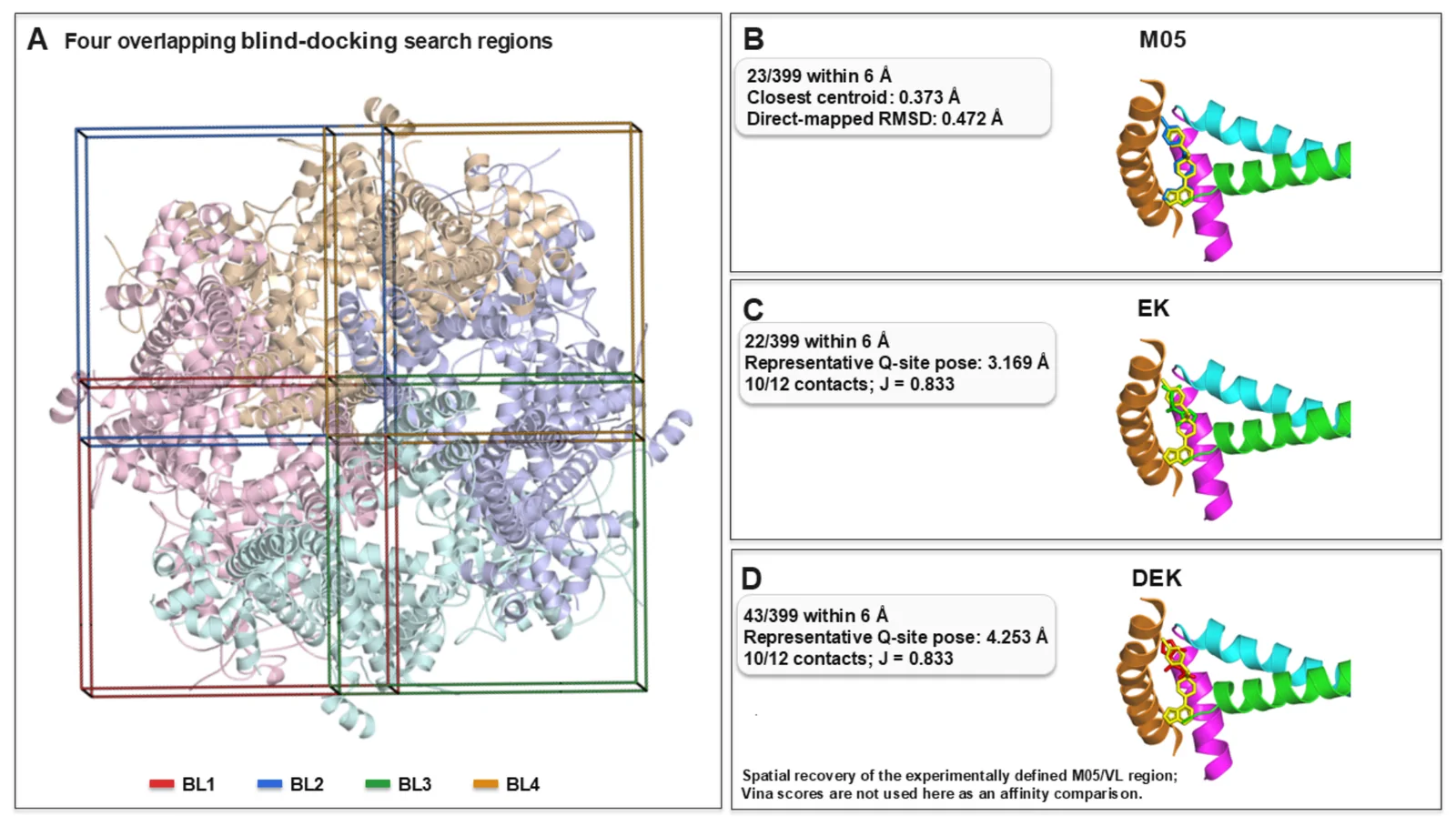
Blind-docking recovery of the experimentally defined M05/VL region and independent localization of EK and DEK. (**A**) Four partially overlapping geometry-derived blind-docking regions spanning the transmembrane domain. (**B**) M05-positive structural control: 23/399 poses were within 6 Å and 96/399 within 10 Å of the nearest symmetry-equivalent experimental M05 centroid; closest centroid displacement 0.373 Å and direct mapped heavy-atom RMSD 0.472 Å. (**C**) EK: 22/399 poses within 6 Å and 63/399 within 10 Å; representative VL-localized pose 3.169 Å from the nearest M05 centroid with 10/12 reference contacts (J = 0.833). (**D**) DEK: 43/399 poses within 6 Å and 82/399 within 10 Å; representative VL-localized pose 4.253 Å from the nearest M05 centroid with 10/12 contacts (J = 0.833).

**Figure 6:**
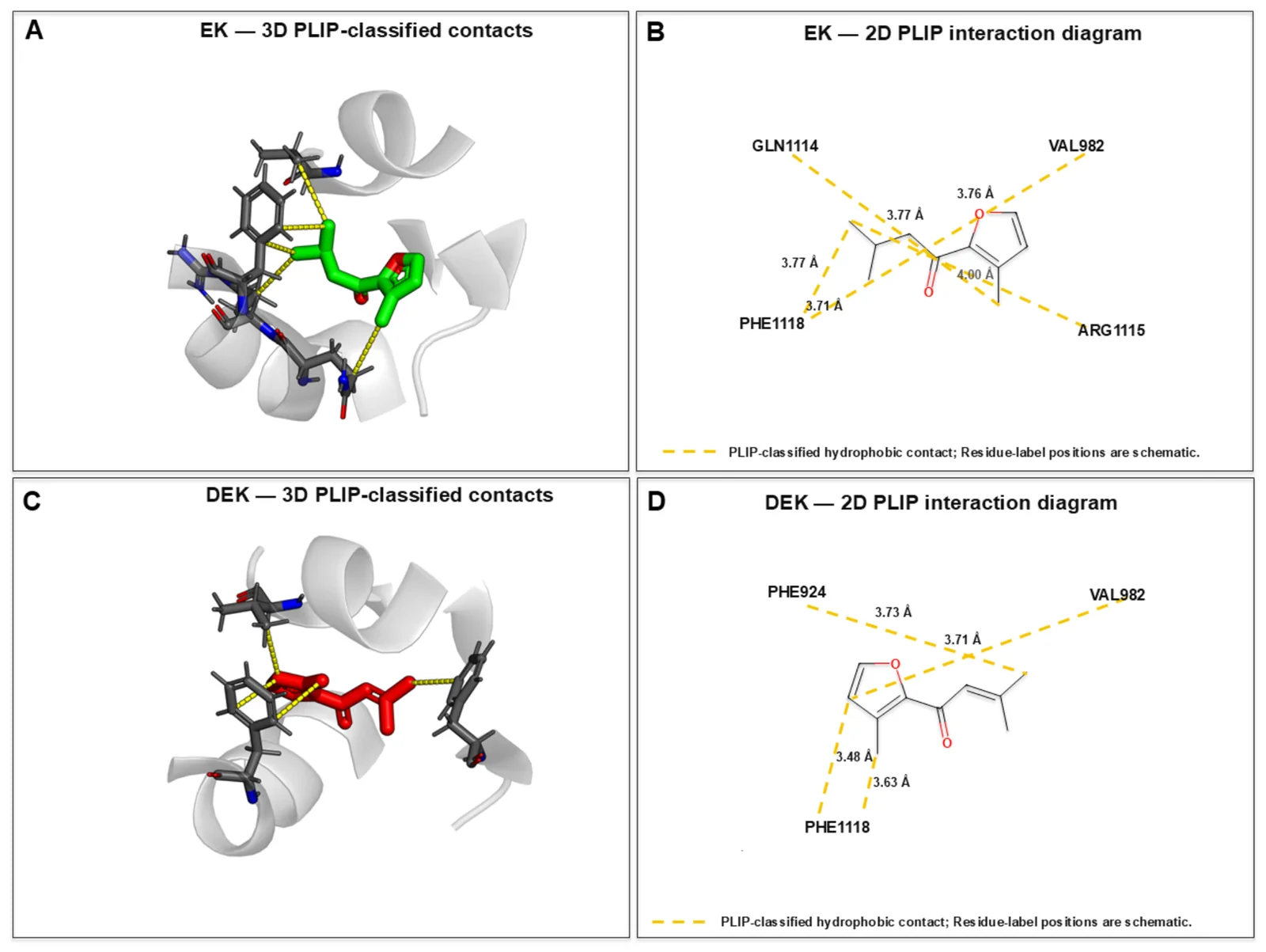
Reference-compatible EK and DEK poses and their PLIP-classified interactions within the TRPM7 VL region. (**A**) RICS-selected EK pose with five PLIP-classified hydrophobic contacts involving VAL982, GLN1114, ARG1115 and PHE1118, including two contacts with PHE1118. (**B**) Corresponding 2D PLIP interaction diagram for EK. (**C**) RICS-selected DEK pose with four hydrophobic contacts involving PHE924, VAL982, and PHE1118, including two contacts with PHE1118. (**D**) Corresponding 2D PLIP interaction diagram for DEK. Both poses reproduced 10/12 M05 reference contacts (J = Recovery = 0.833) without additional contacts; SER921 and TYR923 were not recovered.

**Figure 7:**
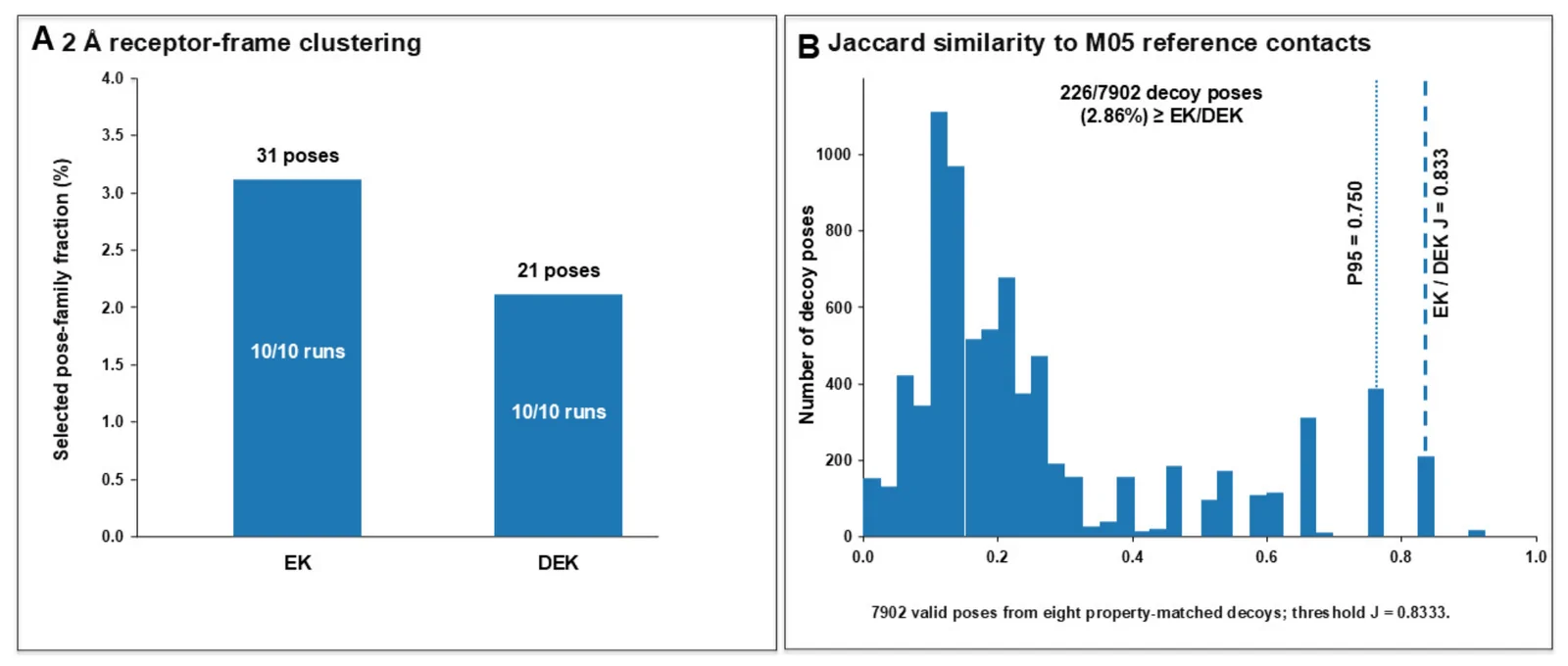
Focused docking, recurrence, and decoy specificity control. (**A**) Pose families containing the final RICS-selected EK and DEK representatives after 2 Å heavy-atom receptor-frame clustering contained 31/998 EK poses (3.11%) and 21/997 DEK poses (2.11%) and were recovered in all 10 independent docking runs. Recurrence reflects stochastic reproducibility, not physical occupancy. (**B**) Jaccard similarity to the experimental M05 contact fingerprint among 7902 valid poses from eight decoys matched by physicochemical properties. EK and DEK representatives had J = 0.8333; the decoy 95th percentile was 0.750, and 226 of 7902 decoy poses (2.86%) reached or exceeded the EK and DEK value, showing that M05-reference contact compatibility alone is not ligand specific.

**Figure 8:**
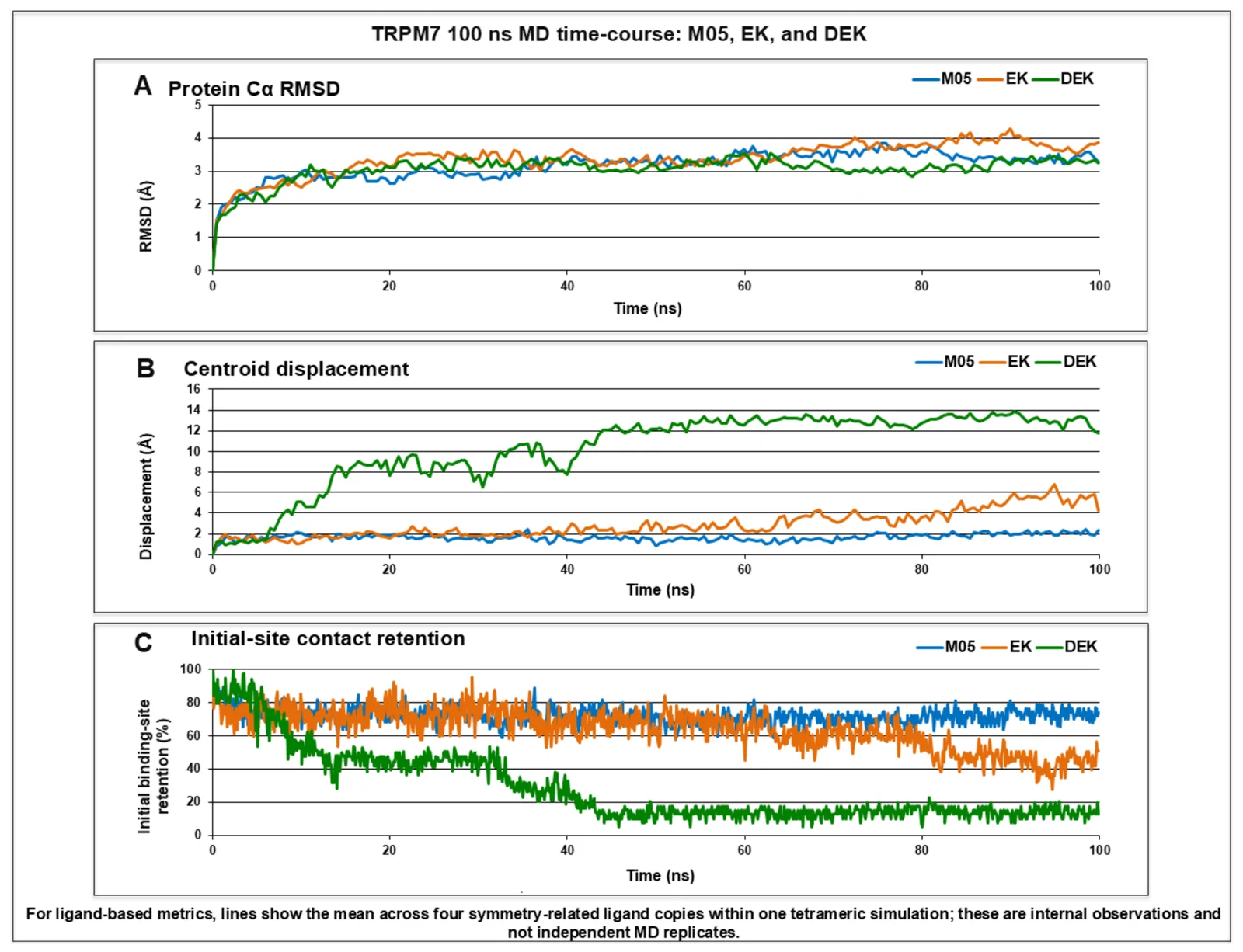
Structural and positional dynamics of M05, EK and DEK during 100-ns explicit-membrane molecular dynamics simulations. (**A**) Protein Cα RMSD over the production trajectory. (**B**) Ligand centroid displacement from the initial position after global alignment on protein Cα atoms. (**C**) Initial-site contact retention, defined as the percentage of frame-0 contacting residues remaining within 4.0 Å of the ligand. Lines in panels (**B**,**C**) are means across the four symmetry-related ligand instances within one tetrameric simulation; these are internal observations rather than independent MD replicates. M05 showed the strongest retention; EK showed progressive and heterogeneous contact loss, and DEK showed substantially greater disruption of the initial interaction environment.

## Data Availability

The original contributions presented in this study are included in the article/Supplementary Material. Further inquiries can be directed to the corresponding author.

## Supplementary Materials

The following supporting information can be downloaded at: https://www.mdpi.com/article/10.3390/ph19101530/s1, Figure S1: Spatial comparison of representative ligand poses in the TRPM7 vanilloid-like region defined by M05; Figure S2: Poses compatible with the reference and PLIP interactions of natural product and simplified structural comparators in the TRPM7 vanilloid-like region defined by M05; Figure S3: Poses compatible with the reference and PLIP interactions of antiarrhythmic comparators in the TRPM7 vanilloid-like region defined by M05; Figure S4: RICS-selected poses of TRPM7 structural and functional comparators in the vanilloid-like region defined by M05; Figure S5: Separate structural analysis of 2-APB in the TRPM7 vanilloid-like region defined by M05; Figure S6: Contact fingerprint heatmap of representative poses relative to the TRPM7 vanilloid-like region defined by M05; Figure S7: Ligand heavy-atom RMSD during 100 ns molecular dynamics in an explicit membrane; Figure S8: Evolution of the ligand contact environment during 100 ns molecular dynamics in an explicit membrane; Table S1: Reference-interaction compatibility of RICS-selected focused-docking poses in the M05-defined TRPM7 VL region; Table S2: Sensitivity of RICS pose selection to the within-ligand Vina-support weight in the nine-ligand focused-docking manuscript set; Table S3: Validation and sampling summary of the revised TRPM7 structural-docking workflow; Table S4: Ligand-instance-resolved quantitative summary of 100-ns explicit-membrane molecular dynamics simulations.

## Abbreviations

The following abbreviations are used in this manuscript:

EK	Elsholtzia ketone
DEK	Dehydroelsholtzia ketone
EO	Essential oil
MD	Molecular dynamics
RICS	Reference Interaction Compatibility Score
TRPM	Transient receptor potential melastatin
5-MF	5-Methylfurfural
2-FMK	2-Acetylfuran
wordVL	vanilloid-like
