# Supplementary material for: Structural Compatibility and Dynamic Behaviour of Elsholtzia Ketones in the CCT128930-Defined TRPM7 Vanilloid-like Region
Source: Pharmaceuticals (Basel). 2026 Sep 27;19(10):1530. doi: 10.3390/ph19101530 (PMC13708375; doi:10.3390/ph19101530)
Supplement: Supplementary file 1 [file pharmaceuticals-19-01530-s001.zip › pharmaceuticals-4554149-supplementary.pdf]

Supplementary Materials

Structural Compatibility and Dynamic Behaviour of Elsholtzia Ketones in the CCT128930-Defined TRPM7 Vanilloid-Like Region, by Jonas Jurevičius et al.

Reference definition. Throughout the Supplementary Materials, CCT128930/M05 in PDB 8W2L is the experimental structural reference. The 12-residue M05 geometric contact fingerprint comprises SER921, ASP922, TYR923, PHE924, ASP978, ALA981, VAL982, MET991, TRP1111, GLN1114, ARG1115, and PHE1118, using a minimum ligand-protein heavy-atom distance of 4.0 Å. Reference Interaction Compatibility Score (RICS) is used only to select representative poses within each ligand ensemble. It is not a measure of binding free energy, affinity, potency, selectivity, or biological activity.

Supplementary Figures

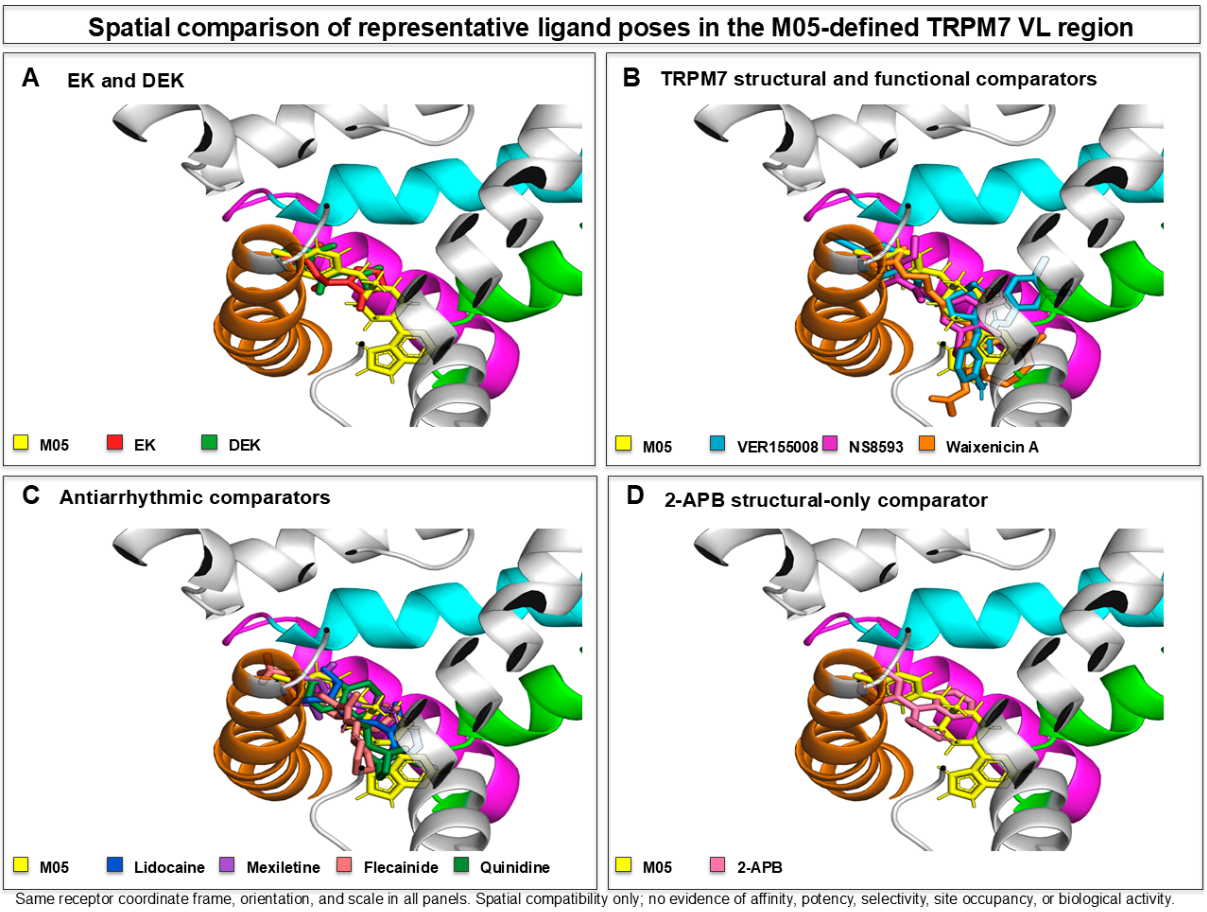

Figure S1. Spatial comparison of representative ligand poses in the TRPM7 vanilloid-like region defined by M05. (A) Representative EK (red) and DEK (green) poses selected by RICS are compared with the minimised experimental M05 reference (yellow). (B) Representative poses of the TRPM7 structural and functional comparators VER155008 (cyan), NS8593 (magenta), and Waixenicin A (orange) are shown relative to M05. (C) Representative poses of the antiarrhythmic comparators lidocaine (blue), mexiletine (violet), flecainide (salmon), and quinidine (dark green) are shown in the same structural reference frame. (D) The representative 2-APB pose (pink), obtained separately by focused AutoDock4 docking, is shown only as a structural comparator relative to M05. All panels use the same receptor coordinate frame, viewing orientation, and scale. The overlays show spatial compatibility with the VL region defined by M05.

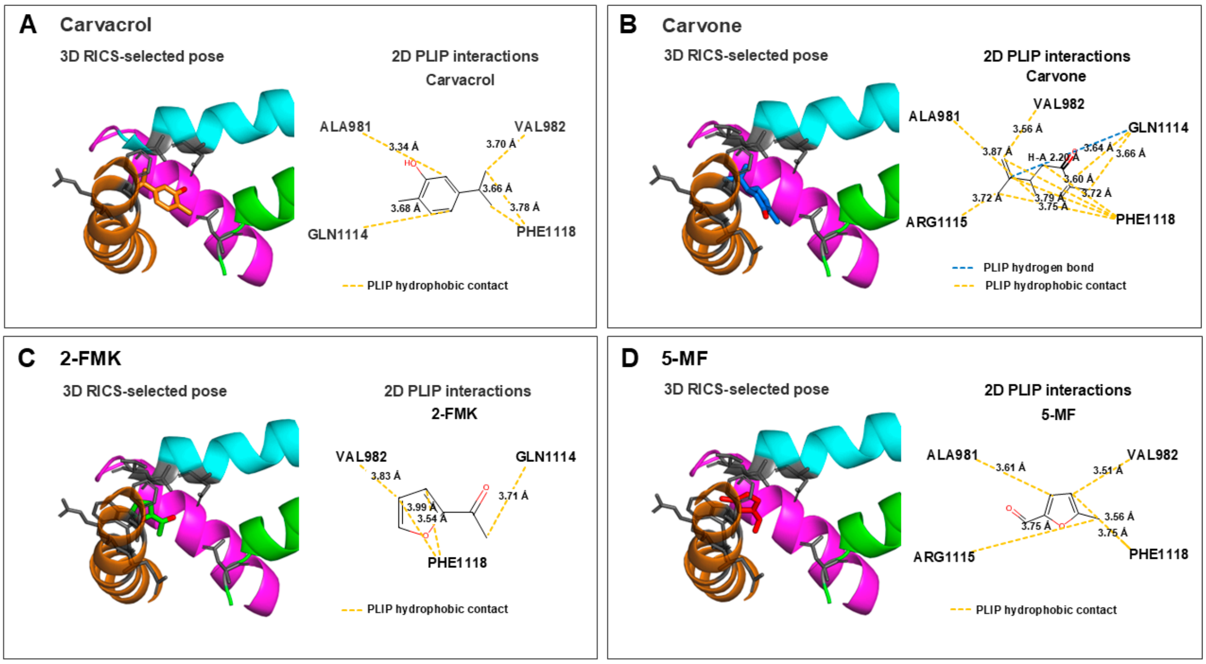

RICS-selected focused-docking representatives; interactions are PLIP-classified. All 3D panels show the same structural orientation.

Figure S2. Poses compatible with the reference and PLIP interactions of natural product and simplified structural comparators in the TRPM7 vanilloid-like region defined by M05. Representative focused docking poses selected by RICS are shown for (A) carvacrol, (B) carvone, (C) 2-FMK, and (D) 5-MF. For each compound, the left panel shows the 3D position of the selected pose in the VL region, and the right panel shows the corresponding 2D PLIP interaction diagram. The 3D panels use the same receptor orientation to facilitate spatial comparison. PLIP annotations describe geometrically classified interactions in the selected docking poses; they are not experimentally validated contacts and do not establish dynamic persistence.

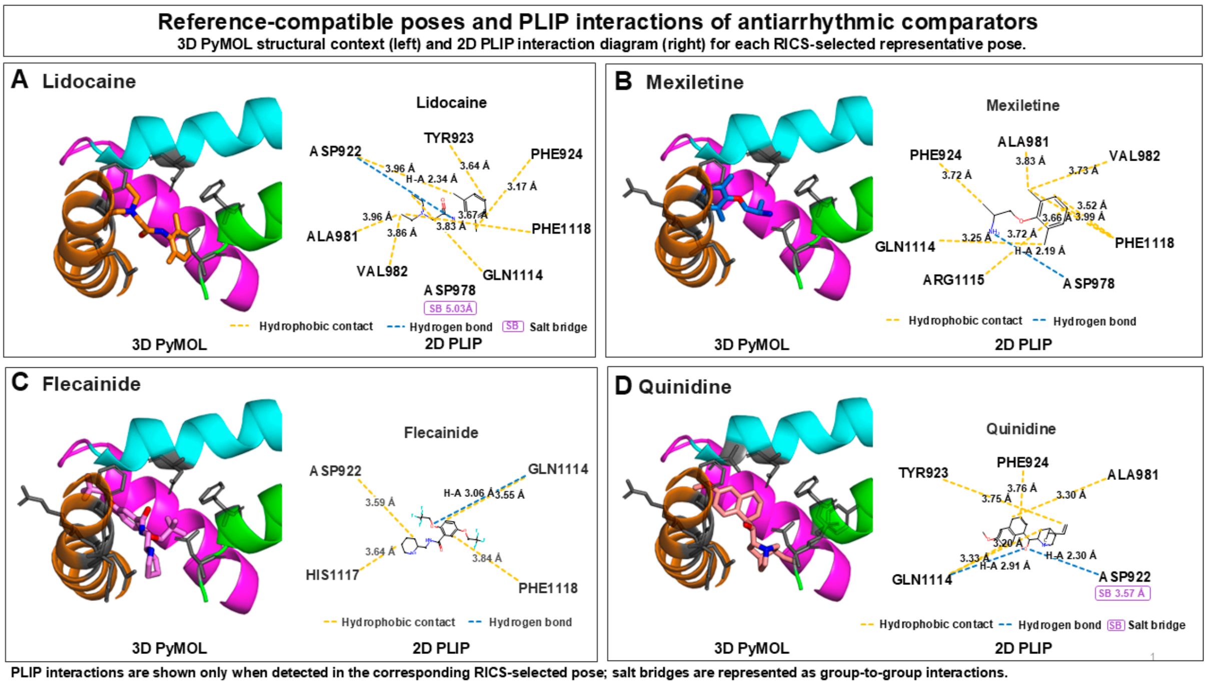

Figure S3. Poses compatible with the reference and PLIP interactions of antiarrhythmic comparators in the TRPM7 vanilloid-like region defined by M05. Representative focused docking poses selected by RICS are shown for (A) lidocaine, (B) mexiletine, (C) flecainide, and (D) quinidine. For each compound, the left panel shows the 3D structural context of the selected pose, and the right panel shows the corresponding 2D PLIP interaction diagram. PLIP interactions are displayed only when detected for the selected pose; salt bridges, where present, are represented as group-to-group interactions. These annotations describe geometric features of docking poses and should not be interpreted as experimentally validated or dynamically persistent contacts. RICS is used for pose selection within each ligand ensemble and is not a measure of affinity, potency, selectivity, or biological activity.

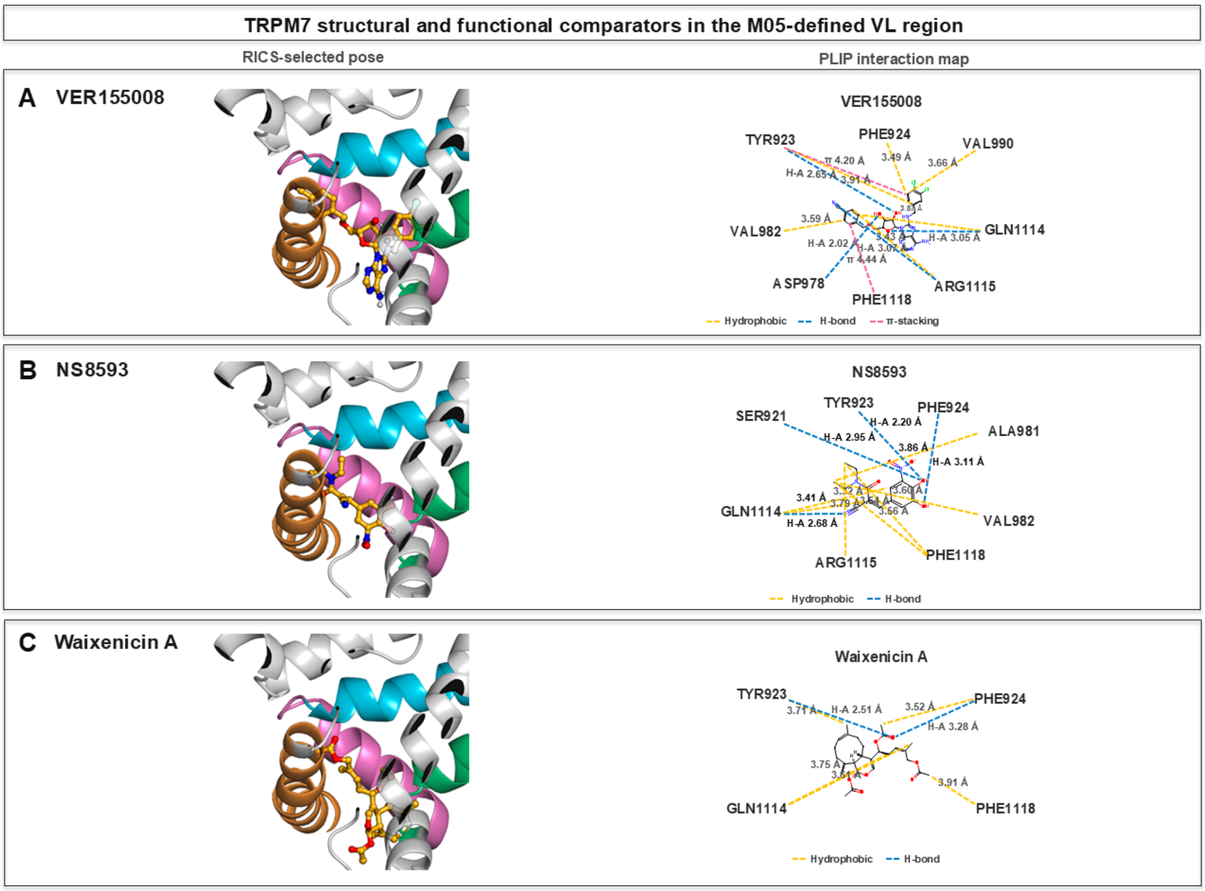

3D: RICS-selected poses in the minimized TRPM7 structural context. 2D: PLIP-classified interactions. Structural comparison only; not evidence of affinity, potency, selectivity, or experimental VL-site occupancy.

Figure S4. RICS-selected poses of TRPM7 structural and functional comparators in the vanilloid-like region defined by M05. (A) VER155008, (B) NS8593, and (C) Waixenicin A. Left panels show representative poses in the minimised TRPM7 VL structural context, while right panels show PLIP-derived 2D interaction maps. VER155008 displayed hydrophobic contacts with TYR923, PHE924, VAL982, VAL990, GLN1114, and ARG1115, hydrogen bonds with TYR923, ASP978, GLN1114, and ARG1115, and π stacking with TYR923 and PHE1118. NS8593 showed hydrophobic interactions involving ALA981, VAL982, GLN1114, ARG1115, and PHE1118, and hydrogen bonds involving SER921, TYR923, PHE924, and GLN1114. Waixenicin A showed hydrophobic contacts with TYR923, PHE924, GLN1114, and PHE1118, and hydrogen bonds involving TYR923 and PHE924. These compounds are presented as structural and functional comparators; the analysis does not establish experimental occupancy of the VL region.

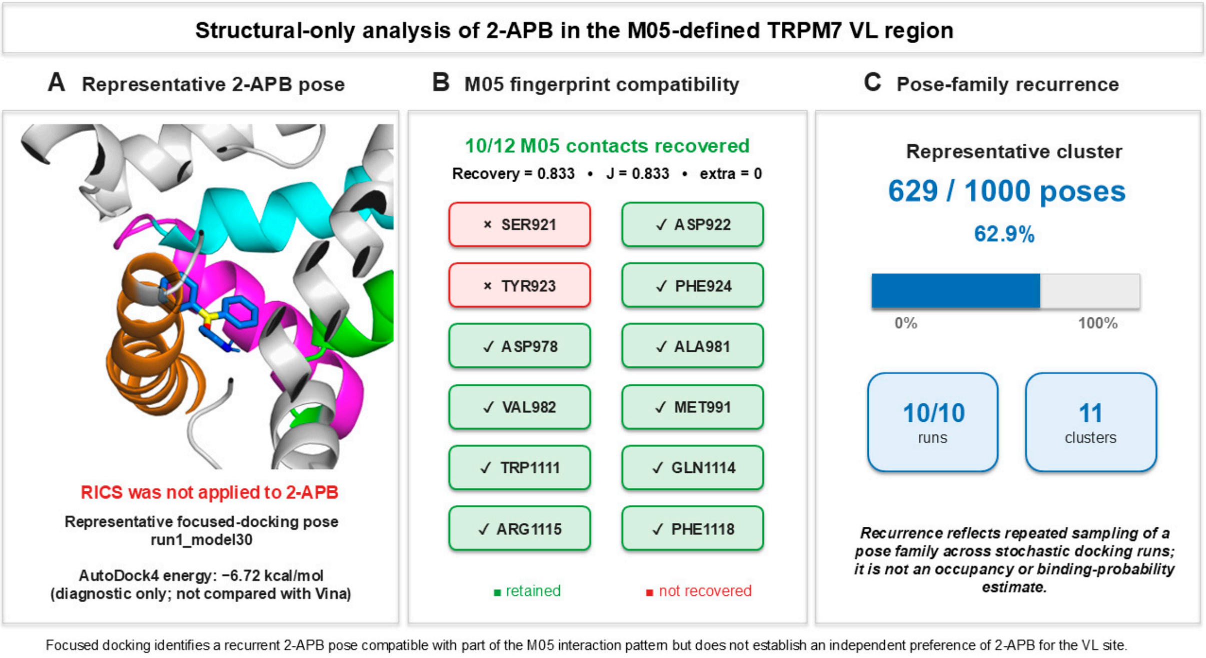

Figure S5. Separate structural analysis of 2-APB in the TRPM7 vanilloid-like region defined by M05. (A) Representative 2-APB pose (run1_model30) obtained by focused AutoDock4 docking in the VL region defined by M05. The AutoDock4 estimated binding energy for this pose was −6.72 kcal/mol and is reported only as a diagnostic docking value; it was not compared with Vina scores for the other ligands. (B) Compatibility of the representative 2-APB pose with the 12-residue M05 interaction-fingerprint. The pose reproduced 10 of 12 M05 contacts (Recovery = 0.833; Jaccard index, J = 0.833) without additional contacts; SER921 and TYR923 were not recovered. (C) Recurrence analysis of 1,000 poses generated in 10 independent stochastic docking runs. At a 2 Å heavy-atom clustering threshold, 11 pose clusters were identified. The cluster containing the representative structural hypothesis comprised 629 of 1,000 poses (62.9%) and was recovered in all 10 runs. Cluster recurrence describes repeated sampling of a geometrically similar pose family and should not be interpreted as site occupancy or binding probability. Because 2-APB was docked directly to the predefined region centred on M05, these results demonstrate structural compatibility with the M05 interaction pattern but do not establish independent preference for the VL site, binding affinity, selectivity, or functional TRPM7 modulation.

Contact-fingerprint heatmap for RICS-selected poses and the 2-APB structural comparator

Ligand	RICS	J	Rec.	SER 921	ASP 922	TYR 923	PHE 924	ASP 978	ALA 981	VAL 982	MET 991	TRP 1111	GLN 1114	ARG 1115	PHE 1118	Extra contacts
2K	0.687	0.667	0.667					•	•	•	•	•	•	•	•	—
5K	0.700	0.667	0.667					•	•	•	•	•	•	•	•	—
CR	0.770	0.833	0.833		•		•	•	•	•	•	•	•	•	•	—
CV	0.762	0.750	0.750		•			•	•	•	•	•	•	•	•	—
EK	0.816	0.833	0.833		•		•	•	•	•	•	•	•	•	•	—
DEK	0.824	0.833	0.833		•		•	•	•	•	•	•	•	•	•	—
NS8593	0.904	1.000	1.000	•	•	•	•	•	•	•	•	•	•	•	•	—
VER155008	0.856	0.923	1.000	•	•	•	•	•	•	•	•	•	•	•	•	VAL990
Waixenicin A	0.831	0.923	1.000	•	•	•	•	•	•	•	•	•	•	•	•	B:ARG731
Lidocaine	0.968	1.000	1.000	•	•	•	•	•	•	•	•	•	•	•	•	—
Mexiletine	0.850	0.833	0.833		•		•	•	•	•	•	•	•	•	•	—
Flecainide	0.864	0.923	1.000	•	•	•	•	•	•	•	•	•	•	•	•	HIS1117
Quinidine	0.900	1.000	1.000	•	•	•	•	•	•	•	•	•	•	•	•	—
2-APB	—	0.833	0.833		•		•	•	•	•	•	•	•	•	•	—

M05 fingerprint contact present; not recovered; 2-APB structural-only contact; *2-APB was docked directly to the M05-centered region and is shown only as a structural comparator.*

RICS was used only for within-ligand pose selection and should not be interpreted as binding affinity, potency, selectivity, or biological activity.

Figure S6. Contact fingerprint heatmap of representative poses relative to the TRPM7 vanilloid-like region defined by M05. The heatmap summarises contacts with the 12-residue interaction-fingerprint defined by the experimental M05 reference: SER921, ASP922, TYR923, PHE924, ASP978, ALA981, VAL982, MET991, TRP1111, GLN1114, ARG1115, and PHE1118. For each ligand, the pose with the highest RICS in the focused docking ensemble is shown. Green cells indicate M05 fingerprint contacts reproduced by the representative pose, while gray cells indicate reference contacts that were not recovered. Additional contacts outside the 12-residue M05 fingerprint are listed separately. RICS, Jaccard similarity (J), and Recovery are reported for each selected pose. The 2-APB pose is shown separately in blue as a structural comparator because it was docked directly to the predefined region centred on M05 and was not subjected to RICS selection. Its representative pose reproduced 10 of 12 M05 contacts (J = Recovery = 0.833) without additional contacts.

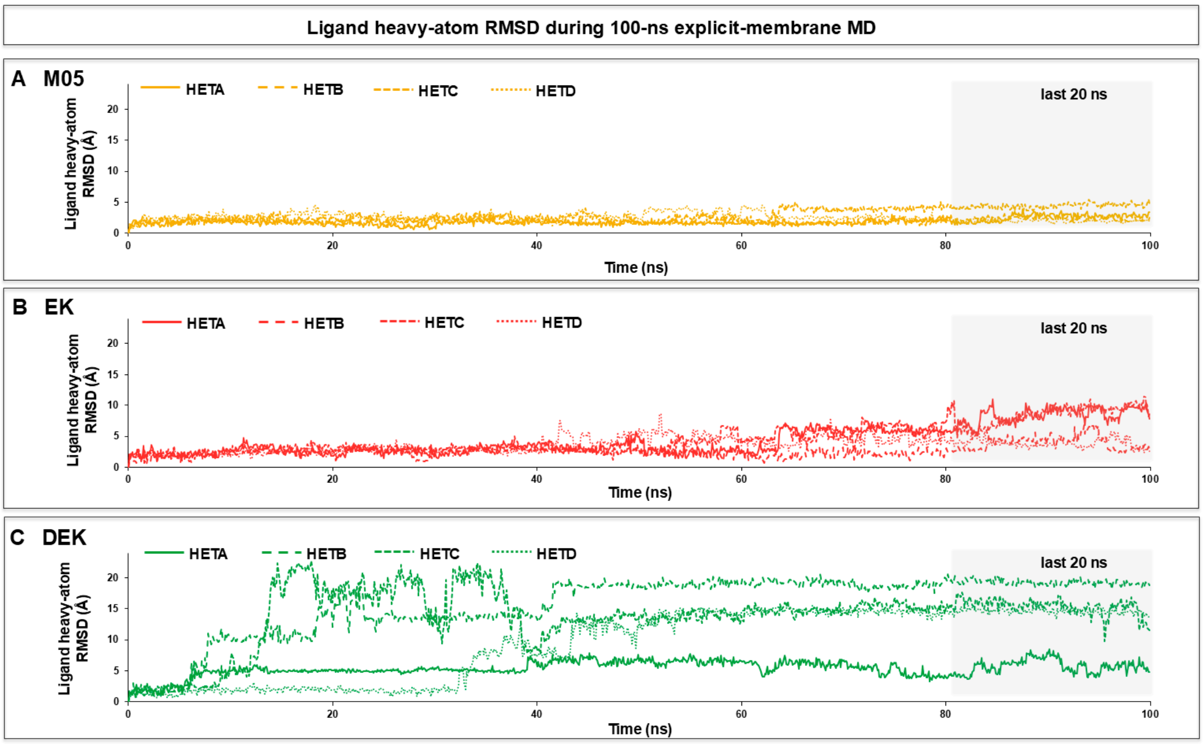

Figure S7. Ligand heavy-atom RMSD during 100 ns molecular dynamics in an explicit membrane. Ligand heavy-atom RMSD trajectories are shown for (A) M05, (B) EK, and (C) DEK over 100 ns. HETA–HETD denote the four symmetry-related ligand copies in the tetrameric TRPM7 simulation system. They are internal observations, not independent simulation replicates. All panels use the same 0–24 Å RMSD scale to allow direct comparison with the initial ligand configuration. The shaded region denotes the final 20 ns used for late trajectory summary statistics. M05 showed comparatively limited deviation, EK displayed heterogeneous intermediate behaviour, and DEK showed substantially larger differences among ligand copies. Ligand RMSD describes deviation from the initial configuration.

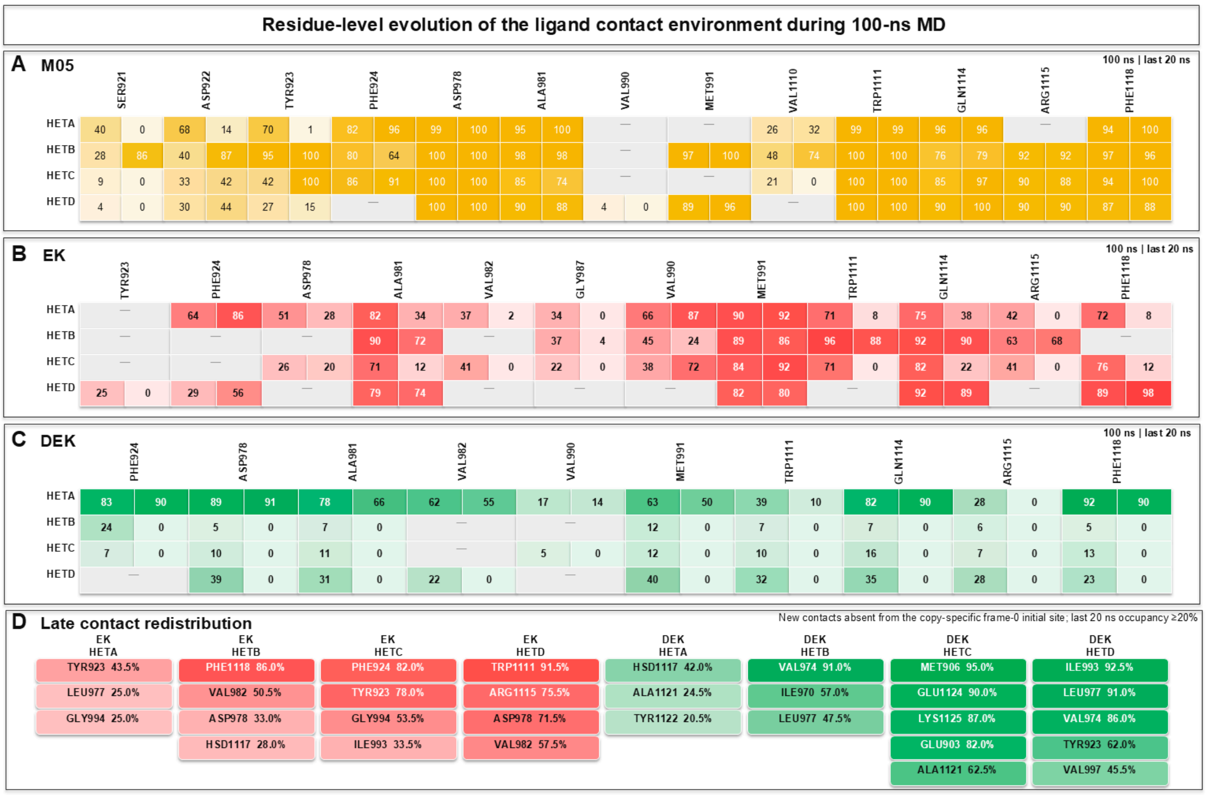

Figure S8. Evolution of the ligand contact environment during 100 ns molecular dynamics in an explicit membrane. Initial site residue contact occupancies are shown for (A) M05, (B) EK, and (C) DEK. For each HETA–HETD ligand copy, the left half of each cell denotes occupancy over the complete 100 ns trajectory and the right half denotes occupancy during the final 20 ns. Contacts were defined using a minimum ligand-protein heavy-atom distance of ≤4.0 Å. Initial site residues were determined independently for each symmetry-related ligand copy from frame 0. Gray cells marked with a dash therefore indicate that the residue was not part of that copy's initial site and are not zero-occupancy measurements. (D) New late contacts absent from the initial site at frame 0 are shown when their final 20 ns occupancy was ≥20%. HETA–HETD are symmetry-related ligand copies within one tetrameric simulation system and are internal observations rather than independent simulation replicates. Contact occupancy describes geometric persistence of a local contact environment.

Supplementary Tables

Table S1. Reference-interaction compatibility of RICS-selected focused-docking poses in the M05-defined TRPM7 VL region.

Ligand	Category	RICS	Jaccard	Recovery (%)	Shared M05 contacts	Additional contacts (n)
EK	*Elsholtzia ciliata* ketone	0.816	0.833	83.3	10/12	0
DEK	*Elsholtzia ciliata* ketone	0.824	0.833	83.3	10/12	0
Carvacrol	Natural-product comparator	0.770	0.833	83.3	10/12	0
Carvone	Natural-product comparator	0.762	0.750	75.0	9/12	0
2-FMK	Simplified furan analogue	0.687	0.667	66.7	8/12	0
5-MF	Simplified furan analogue	0.700	0.667	66.7	8/12	0
NS8593	TRPM7 structural/functional comparator	0.904	1.000	100.0	12/12	0
VER155008	TRPM7 structural/functional comparator	0.856	0.923	100.0	12/12	1
Waixenicin A	TRPM7 functional comparator	0.831	0.923	100.0	12/12	1
Lidocaine	Class IB antiarrhythmic comparator	0.968	1.000	100.0	12/12	0
Mexiletine	Class IB antiarrhythmic comparator	0.850	0.833	83.3	10/12	0
Flecainide	Class IC antiarrhythmic comparator	0.864	0.923	100.0	12/12	1
Quinidine	Class IA antiarrhythmic comparator	0.900	1.000	100.0	12/12	0

RICS (Reference Interaction Compatibility Score) was used only for within-ligand representative-pose selection and is defined as 0.90J + 0.10A, where J is the Jaccard similarity to the 12-residue experimental M05 reference-contact fingerprint and A is a within-ligand normalised Vina support term. Recovery is the fraction of the 12 M05 reference contacts reproduced by the selected pose. Waixenicin A is presented as a functional TRPM7 comparator rather than as an experimentally resolved VL-site ligand. Class I antiarrhythmics are pharmacological comparators and are not established TRPM7-targeting drugs. 2-APB is excluded because it was analysed separately with AutoDock4 as a structural-only comparator.

Table S2. Sensitivity of RICS pose selection to the within-ligand Vina-support weight in the nine-ligand focused-docking manuscript set.

Affinity-support weight (A)	Jaccard weight (J)	Representative poses retained	Mean Top-10 overlap	Mean Top-20 overlap	Spearman rho
0.05	0.95	9/9	9.56/10	20.00/20	0.9899
0.10	0.90	9/9	9.56/10	19.00/20	0.9716
0.15	0.85	9/9	9.56/10	17.44/20	0.9423
0.20	0.80	8/9	8.56/10	14.56/20	0.9015
0.25	0.75	8/9	7.78/10	12.44/20	0.8551

Sensitivity testing varied only the relative contribution of Jaccard similarity (J) and the within-ligand Vina-support term (A), with $J + A = 1.00$. The final RICS formulation uses $A = 0.10$ and $J = 0.90$. At $A = 0.05$-0.15, all 9/9 representative poses were retained; at $A \geq 0.20$, one representative pose changed. $A = 0.10$ was selected as a conservative compromise: it provides a limited Vina-derived ranking contribution without changing any representative pose selected by interaction-pattern similarity, while preserving high agreement in the upper-ranked pose sets. Top-10 and Top-20 overlap values are averages across the nine ligand ensembles. Spearman rho summarises rank-order concordance relative to the J-dominated reference ranking. This sensitivity analysis applies to the locked nine-ligand manuscript-focused ensemble (8,934 poses; ten independent docking runs per ligand). It should not be extrapolated to earlier broader exploratory ligand panels.

Table S3. Validation and sampling summary of the revised TRPM7 structural-docking workflow.

Stage	System	Sampling / criterion	Key quantitative result	Purpose / interpretation
Restrained minimisation	8W2L + M05	Explicit-membrane OpenMM L-BFGS minimisation; structural preservation assessed after protein alignment	Global Cα RMSD 0.0485 Å; VL12 heavy-atom RMSD 0.0864 Å; M05 heavy-atom RMSD 0.0918 Å; 12/12 VL contacts retained	Confirms that system relaxation preserved the experimentally resolved M05/VL structural environment
M05 redocking validation	M05/CCT128930	AutoDock Vina 1.2.7; 10 independent runs × 100 analysed poses; native-like criterion: direct heavy-atom RMSD ≤2.0 Å	Native-like pose recovered in 10/10 runs; best RMSD 0.567 Å; median Vina rank 9 (range 8–10); 13/1000 native-like poses	Validates sampling of the experimental geometry while showing that Vina rank 1 is not a reliable native-pose selector
Unbiased blind docking	M05	Four partially overlapping geometry-derived search regions; 399 valid poses	23/399 poses ≤6 Å and 96/399 ≤10 Å from nearest experimental M05 centroid; closest centroid 0.373 Å; direct RMSD 0.472 Å	Demonstrates independent spatial recovery of the experimentally defined VL region
Unbiased blind docking	EK	Same four-region blind-search strategy; 399 valid poses	22/399 poses ≤6 Å and 63/399 ≤10 Å; mean J = 0.669; best J = 0.833; best-contact solution reproduced 10/12 M05 contacts with 0 extra contacts	Shows that EK can sample the VL region without a ligand-centred search constraint
Unbiased blind docking	DEK	Same four-region blind-search strategy; 399 valid poses	43/399 poses ≤6 Å and 82/399 ≤10 Å; all 4 tile rank-1 poses within 6 Å; mean J = 0.652; best J = 0.833	Shows that DEK can repeatedly sample the VL region without a ligand-centred search constraint
Focused docking + 2 Å clustering	Nine-ligand manuscript set	8,934 poses; 10 independent Vina runs per ligand; RICS-selected pose families clustered at 2.0 Å heavy-atom RMSD in receptor frame	8/9 selected pose families recovered in 10/10 runs; Waixenicin A in 6/10; EK family 31/998 (10/10); DEK family 21/997 (10/10)	Tests stochastic reproducibility of selected structural families; recurrence is not equilibrium occupancy
Property-matched decoy control	8 decoys	80 docking files; 7,902/8,000 valid poses; same M05-reference contact framework	Jaccard mean 0.2645; median 0.1875; P95 0.750; P99 0.8333; maximum 0.9167	Defines the specificity limit: EK/DEK-like M05-reference similarity is not ligand-specific
2-APB structural-only focused docking	2-APB	AutoDock4 with boron-specific parameterisation; 10 independent runs × 100 GA runs = 1,000 poses; 2.0 Å symmetry-aware clustering	Representative run1_model30: J = Recovery = 0.8333, 10/12 contacts, 0 extras; 11 clusters; representative family 629/1000 (62.9%); recurrence 10/10 runs	Demonstrates reproducible structural compatibility within the predefined VL region, not independent VL-site preference

J denotes full-contact Jaccard similarity to the experimentally resolved M05/CCT128930 reference fingerprint. Blind-docking distances are measured to the nearest symmetry-equivalent experimental M05 centroid. Focused-docking cluster recurrence reflects stochastic reproducibility across independent docking runs and must not be interpreted as equilibrium occupancy or binding probability. 2-APB was analysed separately with AutoDock4 because of boron parameterisation and was not assigned a RICS value. Its AutoDock4 energy is diagnostic only and is not quantitatively compared with Vina energies.

Table S4. Ligand-instance-resolved quantitative summary of 100-ns explicit membrane molecular dynamics simulations.

Ligand	Instance	Ligand RMSD, full trajectory (Å)	Ligand RMSD, last 20 ns (Å)	Initial-site contact retention, full (%)	Initial-site contact retention, last 20 ns (%)	Centroid displacement, last 20 ns (Å)
M05	HETA	1.87 ± 0.54	2.57 ± 0.57	77.0	63.8	1.76 ± 0.55
M05	HETB	2.04 ± 0.43	2.36 ± 0.30	79.2	89.6	1.87 ± 0.35
M05	HETC	2.95 ± 1.08	4.43 ± 0.36	67.7	71.9	3.18 ± 0.35
M05	HETD	2.55 ± 0.65	2.03 ± 0.24	64.6	65.5	1.24 ± 0.35
EK	HETA	4.39 ± 2.45	8.35 ± 1.29	62.3	34.7	6.98 ± 1.79
EK	HETB	2.67 ± 0.97	3.93 ± 1.14	73.2	61.7	2.66 ± 1.15
EK	HETC	4.59 ± 2.47	8.48 ± 1.56	55.4	23.1	7.47 ± 1.50
EK	HETD	3.65 ± 1.26	3.93 ± 0.74	66.0	66.2	2.81 ± 0.77
DEK	HETA	5.40 ± 1.27	5.74 ± 1.12	63.2	55.6	4.95 ± 1.12
DEK	HETB	15.74 ± 4.92	19.28 ± 0.45	n.d.	0.0	18.72 ± 0.47
DEK	HETC	13.88 ± 4.85	15.34 ± 1.23	n.d.	0.1	14.81 ± 1.24
DEK	HETD	9.48 ± 5.74	14.59 ± 0.42	n.d.	0.0	14.17 ± 0.42

RMSD and centroid displacement are reported as mean ± SD after global protein Cα alignment. Last 20 ns denotes 80–100 ns. Initial-site contact retention is the percentage of the copy-specific frame-0 protein residues (defined by a minimum ligand-protein heavy-atom distance ≤4.0 Å) retained during the indicated interval. One 100-ns explicit-membrane trajectory was analysed for each ligand system. HETA–HETD are symmetry-related ligand copies within one tetrameric simulation and are internal observations, not independent MD replicates. The metrics describe positional persistence and evolution of the local contact environment. n.d. = the exact full-trajectory initial-site contact-retention value was not retained in the validated analysis summary.
